# Association of sugar intake from different sources with incident depression in the prospective cohort of UK Biobank participants

**DOI:** 10.1007/s00394-022-03022-7

**Published:** 2022-10-07

**Authors:** Anna Kaiser, Sylva M. Schaefer, Inken Behrendt, Gerrit Eichner, Mathias Fasshauer

**Affiliations:** 1 Institute of Nutritional Science, Justus-Liebig University of Giessen, 35390 Giessen, Germany; 2 Mathematical Institute, Justus-Liebig University of Giessen, Giessen, Germany; 3 Department of Internal Medicine (Endocrinology, Nephrology, and Rheumatology), University of Leipzig, Leipzig, Germany

**Keywords:** Carbohydrates, Depression, Metabolic syndrome, Sugar, UK Biobank

## Abstract

**Purpose:**

To elucidate the association of different sources of free sugars (FS) and intrinsic sugars with depression risk in the prospective population-based UK Biobank cohort.

**Methods:**

Sugar consumption was assessed in 188,426 participants (age range: 39–72 years, 54.4% female) with at least one web-based dietary questionnaire (Oxford WebQ). The hazard ratios (HR) for incident depression were assessed with Cox proportional hazard regression models including sugar intake from different sources as penalized cubic splines to allow non-linear predictor effects. Over a mean follow-up of 12.3 (standard deviation 1.8) years, 5410 incident depression cases occurred.

**Results:**

FS intake was significantly associated with depression risk in an ascending approximately linear way with the lowest HR observed at 9% total energy (%E). In contrast, consumption of intrinsic sugars was not significantly related with incident depression. FS in beverages were significantly associated with depression risk in an ascending approximately linear way with the lowest HR at 4%E whereas no association was found for FS in solids. Concerning beverage types, FS in soda/fruit drinks, milk-based drinks, and tea/coffee were significantly and positively related to depression risk whereas the association was U-shaped for juice. Major findings were robust in sensitivity analyses.

**Conclusion:**

Only some sources of FS are positively associated with incident depression. Public health initiatives targeting FS subtypes might be most effective concerning depression risk if focused on the reduction of sugary beverages and more specifically soda/fruit drinks, milk-based drinks, and tea/coffee.

**Supplementary Information:**

The online version contains supplementary material available at 10.1007/s00394-022-03022-7.

## Introduction

Depression is one of the most common psychiatric conditions affecting about 3.8% of the total and 5.0% of the adult worldwide population [1]. Female sex and a family history of depression are important non-modifiable risk factors [1, 2]. Furthermore, adverse life events and facets of the metabolic syndrome contribute to the disease and are potentially modifiable [1, 3]. Thus, the risk for depression is twice as high in obese patients as compared to normal-weight controls [3, 4]. In addition, several food items, nutrients, as well as dietary patterns, are associated with brain function and mood disorders [5–7].

Low carbohydrate diets are a popular approach to decrease body weight and improve glucose control, as well as low-grade inflammation [8, 9]. Interestingly, preliminary evidence suggests that they might also improve depressive symptoms [10, 11]. However, various food items need to be excluded when adhering to a low carbohydrate diet limiting the diversity of choices and contributing to poor long-term adherence [12]. In addition, several food items and nutrients positively associated with mental health might also be excluded from the diet, e.g., fruits and vegetables, as well as complex carbohydrates found in legumes and whole grains [6]. Therefore, more recent interventions have focused on reducing specific carbohydrate subtypes with a particular emphasis put on limiting sugars [13, 14]. Sugars are all mono- and disaccharides [15] and they can be divided into free sugars (FS) and intrinsic sugars according to the World Health Organization (WHO) [16]. FS are added to foods by the manufacturer, cook, or consumer, plus sugars naturally present in honey, syrups, and fruit juices [16]. The WHO recommends to limit FS throughout the life course to less than 10% of total energy intake, i.e., 50 g FS per day for a 2000 kcal diet, and optimally to even below 5% [16]. The National Health Service (NHS) is even more restrictive limiting FS consumption to less than 30 g per day for adults [17]. However, the WHO recommendation [16] is based on cohort studies of the association between FS intake and dental caries and does not differentiate between FS sources.

Some studies suggest that higher intake of FS in general [18] and in soda/fruit drinks [19–21] is positively associated with depression risk. Mechanisms by which FS might cause the development of the disease include FS-mediated decreases of brain-derived neurotrophic factor (BDNF) [22] and induction of low-grade inflammation [23], as well as addiction-like effects with signs of behavioral depression and anxiety after sugar withdrawal [24].

No study so far has systematically assessed the link between FS consumption from different sources including FS in beverages and beverage subtypes, as well as FS in solids and solids subtypes, on the one hand and depression risk on the other hand. To address this open point, all major FS sources, which are summarized in Online Resource 1, were assessed within the current study in a large, well-characterized population of 188,426 UK Biobank participants using penalized cubic splines to allow, in particular, non-linear predictor effects. Furthermore, the association between intrinsic sugars, i.e., all sugars that are not FS including sugars from fruit, vegetables, and lactose in dairy products [16], and depression risk was studied for the first time. We hypothesized that the association between FS and incident depression depends on FS source with adverse effects being especially related to beverages and differential associations seen for specific beverage subtypes. Moreover, we hypothesized that high consumption of intrinsic sugars in contrast to FS is not related to depression risk.

## Methods

### Study and participants

The UK Biobank study is the basis for all analyses. Between 2006 and 2010, more than 500,000 participants were recruited across the UK [25]. For the current study, participants who filled out at least one web-based dietary questionnaire for the assessment of previous 24 h dietary intakes (Oxford WebQ) [26] were selected as summarized in Online Resources 2 and 3. The following exclusion criteria were applied: (1) diagnosis of depression before completion of last Oxford WebQ, (2) missing socioeconomic factors (Townsend deprivation index, total household income, ethnic background, highest qualification, or overall health rating), (3) missing data of the physical exam [body mass index (BMI), systolic blood pressure (SBP)], (4) being in the upper 0.1% of total energy and/or carbohydrate intake or total energy intake of 0 kJ/day, (5) malabsorption, and (6) missing lifestyle risk factors (physical activity or smoking status) resulting in a study population of 188,426 participants. The diagnosis malabsorption was assessed at baseline by a verbal interview. Mean (range) age was 56 (39–72) years with 102,575 participants (54.4%) being female. The UK Biobank study was approved by the North West Multicentre Research Ethics Committee and written informed consent was obtained from all participants at baseline [25].

### Exposure assessment

Consumption of sugar and sugar subtypes was calculated based on the Oxford WebQ data with methodology similar to recent studies [27, 28]. In brief, energy and total sugar contents were estimated for each Oxford WebQ questionnaire item based on McCance and Widdowson’s The Composition of Foods and its supplements [26], the UK Data Archive Standard Recipes Database [29], and product labels. The procedure to estimate FS content of the food items is based on Wanselius et al. [30] and summarized in Online Resource 4. FS were divided into FS in beverages and FS in solids. The following sugar-containing beverage subtypes were defined: soda/fruit drinks (the following items were assessed during baseline: carbonated (fizzy) drinks, fruit drinks, J_2_0, squash, cordial, excluding low calorie or diet drinks), pure juice (i.e., fruit and vegetable; indicated as “juice” throughout the manuscript), milk-based drinks (i.e., dairy/yogurt-based smoothies, yogurt drinks, flavoured milk or milkshakes, hot chocolate or other milk-based drinks, excluding plain milk), and sugar added to tea/coffee. For tea/coffee, participants could choose the amount of added sugar per drink as half, one, two, or three teaspoon(s), as well as “varied”. This number of teaspoons was multiplied by the total number of cups of coffee and tea consumed, respectively. Since one teaspoon was the most commonly chosen portion size for added sugar in tea/coffee, this amount was set if participants indicated “varied”. The amount of sugar in tea/coffee was reported in the Oxford WebQ on each occasion the questionnaire was filled out. In total, “varied” was indicated on at least one occasion by 1004 participants for sugar added to tea/coffee. The following sugar-containing solids subtypes were defined: treats (i.e., pastries, candies, chocolate, ice cream, sweetened yoghurt), breakfast cereals (i.e., all food items labelled as “cereal(s)”, porridge, muesli, shreddies; indicated as “cereals” throughout the manuscript), toppings (i.e., table sugar, jam, honey, syrup, peanut butter, chocolate/nut spread, stewed/cooked fruit), and sauces (i.e., all food items labelled as “sauce(s)”, “salad cream”, mayonnaise, ketchup, chutney, salad dressing, pesto, gravy). Standard portion sizes were taken from the UK Food Standards Agency [31] and product labels. For each participant, the intake (g/day) of the specific sugar subtype was calculated by multiplying the frequency of each food item with the estimated content of this sugar subtype in that item in a portion. Intrinsic sugars were calculated as the difference between total sugars and FS. Sugar subtype intake in kJ/day was calculated by multiplying the intake in g/day with 17 kJ/g. Sugar subtype consumption in % total energy (%E) was calculated as follows according to Willett and co-workers [32]: Sugar subtype intake in kJ/day × 100%/total energy in kJ/day. UK Biobank participants could fill out the Oxford WebQ on up to five occasions. For participants who completed more than one questionnaire, the mean %E intake of sugar subtypes was used for all primary analyses.

### Outcome assessment

Linked morbidity data are provided by UK Biobank as the earliest record date and respective health outcome defined with three-character International Statistical Classification of Diseases and Related Health Problems, Tenth Revision (ICD-10) codes [33]. Sources for these morbidity data are self-report at baseline assessment, as well as primary care, inpatient hospital, and death record data [33]. In the current study, the primary outcome was incident depression defined as ICD-10 codes F32 and F33. Follow-up time was calculated by subtracting the date of the baseline assessment from the date of the first diagnosis of depression, loss-to-follow-up, death, or censoring (i.e., December 31, 2021), whichever came first. The shortest duration to diagnosis was used in case of both F32 and F33 diagnoses in a patient.

### Statistical analyses

All data were analysed with R version 4.0.5 [34] as described recently [35, 36]. The hazard ratios (HR) for incident depression were assessed with Cox proportional hazard regression multivariate nutrient density models [32] including %E intake of sugar from different sources and energy intake as penalized cubic splines with their degrees of freedom set to 4. Besides energy intake, models were adjusted for age (split by quintiles), alcohol intake (< 1, 1 to < 8, 8 to < 16, ≥ 16 g/day), BMI (< 18.5, 18.5 to < 25, 25 to < 30, ≥ 30 kg/m^2^), ethnic background (White, group composed of Mixed, Asian, Black, Chinese, and other), general health status (poor, fair, good, excellent), highest qualification (none of the below, national exams at age 16 years, vocational qualifications or optional national exams at ages 17–18 years, professional, College or University), history of mental illness (yes, no), physical activity [metabolic equivalent of task (MET)-minutes per week derived from the Oxford WebQ; split by quintiles], SBP (split by quintiles), sex (female, male), smoking status (never, previous, current occasional, current < 10, 10–14, 15–19, ≥ 20 cigarettes per day), total household income (< 18, 18 to < 31, 31 to < 52, 52 to < 100, ≥ 100 k£, unknown), and Townsend deprivation index (split by quintiles). Hazard proportionality was assessed for each covariate based on scaled Schoenfeld residuals. All covariates violating the proportional hazard assumption significantly after Holm-adjustment for multiple testing were stratified in the final models.

In each analysis, determination of the nadir of the estimated HR as a function of the intake of a sugar subtype in %E was restricted to the range from zero to the 99%-quantile. To simplify presentations, the HR was then rescaled to a nadir of 1. HR with pointwise 95% confidence intervals (CIs) are shown for all Cox proportional hazard regression models. The analysis of each penalized cubic spline is separated into p^lin^ for the linear and p^non−lin^ for the nonlinear effect as described recently [35].

Several sensitivity analyses were run similarly as described in a recent study [37] to check the robustness of the findings. Reverse causation was considered by excluding participants lost to follow-up or diagnosed with depression within 2 years after baseline (landmark analysis) and by excluding participants who had lost weight unintentionally. To remove implausible energy intake data, participants with under-reporting, i.e., < 1.1 × basal metabolic rate—500 kcal, or over-reporting, i.e., > 2.5 × basal metabolic rate + 500 kcal were excluded from the analysis. Basal metabolic rate was defined according to the Oxford equation [38]. To control for unrepresentative consumption data, participants who reported their previous day´s diet as non-typical on at least one occasion were excluded. To assess whether the portion size “varied” for sugar added to tea/coffee affected the results, all participants indicating “varied” on at least one occasion were removed from the analysis. To focus on nutrient intake closest to baseline assessment, analyses were repeated using the first Oxford WebQ questionnaire only. To apply alternative measures for body composition, waist-to-hip ratio (WHR) and height instead of BMI were used. To further control for residual confounding by dietary factors, a diet quality score was included in the analysis combining five dietary components, i.e., fat, fruit, vegetables, red meat, and processed meat consumption as described by Anderson et al. [37]. Minimum and maximum instead of mean sugar subtype and energy intake levels were assessed in two additional sensitivity analyses to consider lowest and highest consumption levels reported. To assess sex-dependent differences, sensitivity analyses were conducted in females and males separately.

A *p* value of < 0.05 was regarded as statistically significant in all analyses. If both p^lin^ and p^non−lin^ were non-significant, no further interpretation of the HR-nadir or other individual HR was performed.

## Results

### Baseline data of UK Biobank participants

Baseline characteristics of the study population in total and in subgroups of FS consumption defined by quintiles are shown in Table 1. Over a mean (standard deviation, SD) follow-up of 12.3 (1.8) years and altogether 2.3 million person-years, a total of 5410 incident depression cases occurred with 3447 in females and 1963 in males.

**Table 1:** Baseline characteristics of the UK Biobank cohort

Parameters	Total cohort (*n* = 188,426)	FS intake (%E) split by quintiles				
		0.0–6.6 (*n* = 37,685)	6.6–9.4 (*n* = 37,685)	9.4–12.1 (*n* = 37,685)	12.1–15.6 (*n* = 37,685)	15.6–78.4 (*n* = 37,686)
Characteristics						
Age (years)	56 (8)	56 (8)	56 (8)	56 (8)	56 (8)	55 (8)
BMI (kg/m^2^)	26.9 (4.6)	27.3 (4.7)	26.9 (4.5)	26.7 (4.4)	26.6 (4.4)	26.9 (4.6)
Ethnic background						
White	180,425 (95.8)	36,130 (95.9)	36,422 (96.6)	36,346 (96.4)	36,250 (96.2)	35,277 (93.6)
Mixed, Asian, Black, Chinese, and other	8001 (4.2)	1555 (4.1)	1263 (3.4)	1339 (3.6)	1435 (3.8)	2409 (6.4)
General health status						
Poor	4733 (2.5)	1023 (2.7)	729 (2.1)	766 (2.0)	858 (2.3)	1307 (3.5)
Fair	31,152 (16.5)	6625 (17.6)	5848 (15.5)	5732 (15.2)	5909 (15.7)	7038 (18.7)
Good	113,757 (60.4)	22,529 (59.8)	22,767 (60.4)	23,110 (61.3)	23,037 (61.1)	22,314 (59.2)
Excellent	38,784 (20.6)	7508 (19.9)	8291 (22.0)	8077 (21.4)	7881 (20.9)	7027 (18.6)
Highest qualification						
None of the below	15,875 (8.4)	3512 (9.3)	2963 (7.9)	2954 (7.8)	2965 (7.9)	3481 (9.2)
National exams at age 16 years	28,442 (15.1)	5772 (15.3)	5480 (14.5)	5559 (14.8)	5542 (14.7)	6089 (16.2)
Vocational qualifications or optional national exams at ages 17–18 years	33,756 (17.9)	6927 (18.4)	6501 (17.3)	6554 (17.4)	6497 (17.2)	7277 (19.3)
Professional	29,230 (15.5)	5655 (15.0)	5694 (15.1)	5927 (15.7)	5985 (15.9)	5969 (15.8)
College or University	81,123 (43.1)	15,819 (42.0)	17,047 (45.2)	16,691 (44.2)	16,696 (44.3)	14,870 (39.5)
History of mental illnesses	3049 (1.6)	565 (1.5)	586 (1.6)	573 (1.5)	590 (1.6)	735 (2.0)
Physical activity (MET-min/week)	4114 (2683)	4044 (2716)	4088 (2586)	4111 (2585)	4141 (2622)	4186 (2891)
SBP (mmHg)	139 (19)	140 (19)	139 (19)	139 (19)	139 (19)	138 (19)
Sex—female	102,575 (54.4)	20,063 (53.2)	20,961 (55.6)	20,832 (55.3)	20,802 (55.2)	19,917 (52.8)
Smoking status						
Never	108,165 (57.4)	19,296 (51.2)	20,991 (55.7)	21,974 (58.3)	22,962 (60.9)	22,942 (60.9)
Previous	67,033 (35.6)	15,388 (40.8)	14,279 (37.9)	13,394 (35.5)	12,340 (32.7)	11,632 (30.9)
Occasional	4510 (2.4)	1096 (2.9)	924 (2.5)	851 (2.3)	795 (2.1)	844 (2.2)
Current < 10 cigarettes per day	2251 (1.2)	468 (1.2)	401 (1.1)	391 (1.0)	457 (1.2)	534 (1.4)
Current 10–14 cigarettes per day	1969 (1.0)	383 (1.0)	352 (0.9)	343 (0.9)	346 (0.9)	545 (1.4)
Current 15–19 cigarettes per day	1735 (0.9)	377 (1.0)	275 (0.7)	300 (0.8)	302 (0.8)	481 (1.3)
Current ≥ 20 cigarettes per day	2763 (1.5)	677 (1.8)	463 (1.2)	432 (1.1)	483 (1.3)	708 (1.9)
Total household income per year (k£)						
< 18	24,782 (13.2)	4781 (12.7)	4582 (12.2)	4673 (12.4)	4981 (13.2)	5765 (15.3)
18 to < 31	40,917 (21.7)	7801 (20.7)	7991 (21.2)	8242 (21.9)	8326 (22.1)	8557 (22.7)
31 to < 52	48,582 (25.8)	9527 (25.3)	9859 (26.2)	9793 (26.0)	9807 (26.0)	9596 (25.5)
52 to < 100	42,586 (22.6)	9046 (24.0)	8902 (23.6)	8601 (22.8)	8370 (22.2)	7667 (20.3)
≥ 100	12,794 (6.8)	2926 (7.8)	2807 (7.4)	2584 (6.9)	2419 (6.4)	2058 (5.5)
Unknown	18,765 (10.0)	3604 (9.6)	3544 (9.4)	3792 (10.1)	3782 (10.0)	4043 (10.7)
Townsend deprivation index	− 1.6 (2.8)	− 1.5 (2.9)	− 1.7 (2.8)	− 1.7 (2.8)	− 1.8 (2.8)	− 1.5 (3.0)
Dietary sugar subtype intake in %E						
Carbohydrates	48.7 (8.1)	44.4 (9.3)	46.8 (7.4)	48.4 (6.8)	50.2 (6.4)	53.9 (6.9)
Total sugars	24.4 (7.5)	18.6 (6.8)	21.7 (5.7)	23.8 (5.4)	26.2 (5.3)	31.6 (6.7)
Intrinsic sugars	13.0 (5.8)	14.4 (6.7)	13.7 (5.7)	13.0 (5.4)	12.5 (5.2)	11.3 (5.4)
FS	11.4 (5.9)	4.2 (1.7)	8.0 (0.8)	10.8 (0.8)	13.7 (1.0)	20.3 (4.9)
FS beverages	4.9 (5.1)	1.0 (1.4)	2.5 (2.1)	3.9 (2.6)	5.8 (3.1)	11.4 (6.5)
Soda/fruit drinks	1.8 (3.8)	0.1 (0.6)	0.5 (1.2)	0.9 (1.8)	1.8 (2.6)	5.6 (6.3)
Juice	2.1 (2.8)	0.5 (1.2)	1.4 (1.8)	2.1 (2.2)	2.7 (2.7)	3.7 (4.2)
Milk-based drinks	0.3 (0.9)	0.1 (0.5)	0.2 (0.7)	0.3 (0.9)	0.4 (1.0)	0.6 (1.3)
Tea/coffee	0.6 (1.7)	0.2 (0.6)	0.3 (1.0)	0.5 (1.3)	0.7 (1.6)	1.4 (2.8)
FS solids	6.5 (3.5)	3.2 (1.8)	5.5 (2.2)	6.8 (2.6)	8.0 (3.1)	8.9 (4.2)
Treats	4.3 (3.0)	2.0 (1.6)	3.6 (2.0)	4.5 (2.4)	5.2 (2.8)	6.1 (3.9)
Cereals	0.5 (0.8)	0.3 (0.7)	0.5 (0.7)	0.5 (0.8)	0.6 (0.8)	0.6 (0.9)
Toppings	1.2 (1.6)	0.4 (0.9)	0.9 (1.4)	1.3 (1.6)	1.6 (1.8)	1.6 (2.0)
Sauces	0.3 (0.4)	0.2 (0.4)	0.3 (0.4)	0.3 (0.4)	0.3 (0.4)	0.3 (0.4)
Other nutrients of interest						
Alcohol (g/day)	17.0 (22.0)	25.0 (28.2)	20.1 (23.1)	16.7 (19.9)	13.6 (17.7)	9.8 (15.7)
Fat (g/day)	78.1 (29.4)	71.7 (28.9)	78.7 (28.5)	81.3 (29.0)	81.6 (29.2)	77.3 (30.1)
Protein (g/day)	74.0 (21.9)	74.8 (24.1)	76.2 (21.6)	75.6 (21.0)	74.1 (20.5)	69.2 (21.5)
Fibre (g/day)	18.8 (7.3)	18.8 (7.9)	19.6 (7.2)	19.4 (7.0)	19.0 (6.8)	17.2 (7.0)
Energy (kJ/day)	8979 (2492)	8275 (2454)	8910 (2369)	9151 (2399)	9291 (2431)	9268 (2652)
Number of Oxford WebQ	2.2 (1.2)	2.0 (1.1)	2.3 (1.2)	2.3 (1.2)	2.3 (1.2)	2.0 (1.1)
Follow-up time between baseline and depression diagnosis (years)	7.4 (3.0)	7.4 (3.1)	7.5 (3.0)	7.5 (3.0)	7.3 (3.0)	7.4 (3.0)

Categorical variables are summarized as frequencies (percentages) and continuous variables as mean (SD)

*FS* free sugars, *MET* metabolic equivalent of task

### FS versus intrinsic sugars

**Fig. 1:**
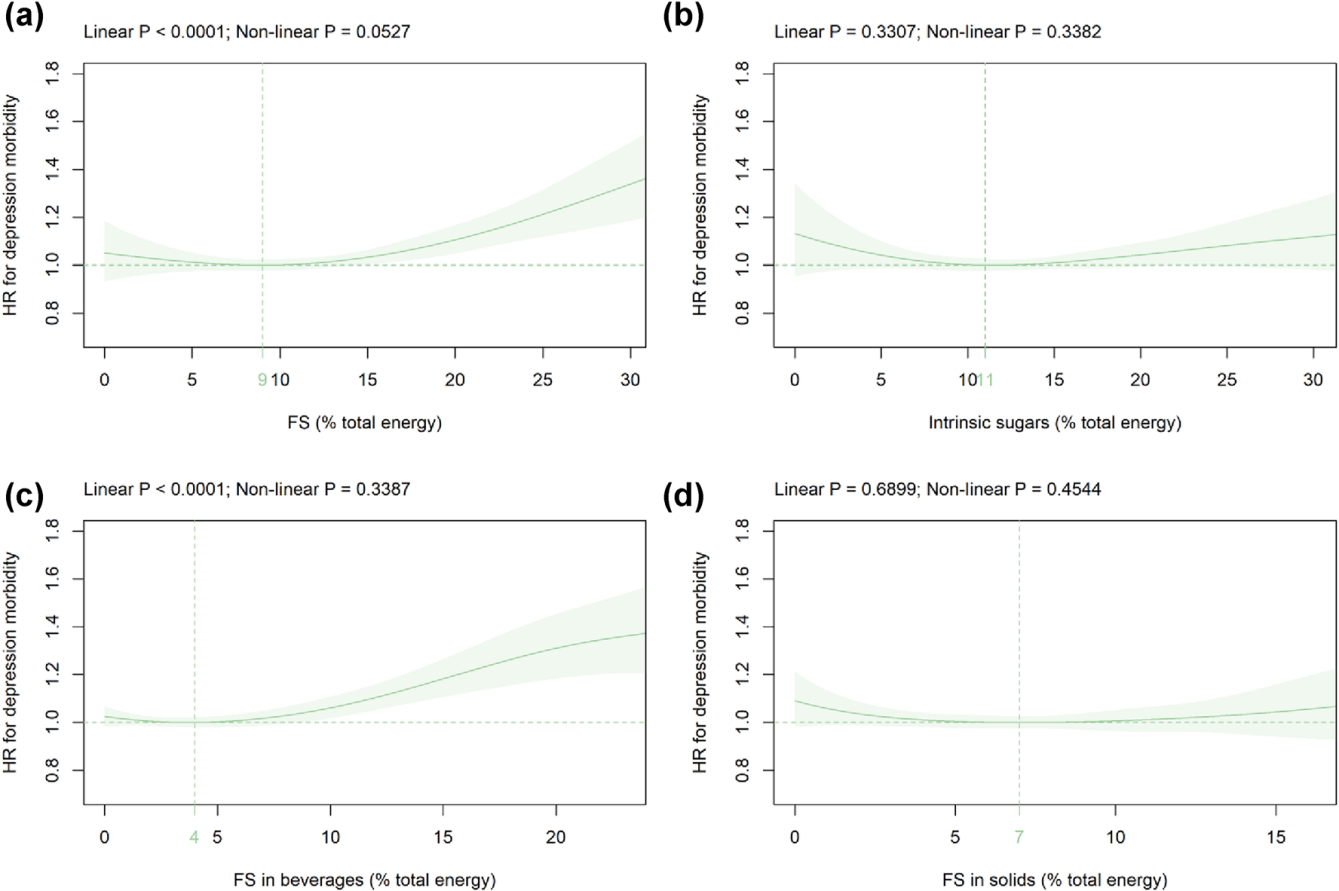
Association of **a** FS, **b** intrinsic sugars, **c** FS in beverages, and **d** FS in solids intake (all %E) with depression risk. Models are adjusted for energy intake, age, alcohol intake, BMI, ethnic background, general health status, highest qualification, history of mental illnesses, physical activity, SBP, sex, smoking status, total household income, and Townsend deprivation index as summarized in the Methods section. Covariates not fulfilling the proportional hazard assumption are stratified. The HR-nadir is indicated in green. *FS* free sugars, *HR* hazard ratio

Mean (SD) consumption of FS and intrinsic sugars was 11.4 (5.9) and 13.0 (5.8) %E, respectively (Table 1). FS intake was significantly associated with the HR for depression in an ascending approximately linear way (Fig. 1a). The HR-nadir for FS was found at 9%E and the HR (CI) increased to 1.11 (1.05 to 1.17) at 20%E (Fig. 1a). In contrast, the intake of intrinsic sugars was not significantly related to depression risk (Fig. 1b).

FS remained significantly related to depression in all sensitivity analyses (Online Resource 5a–16a). The HR-nadir decreased to 0%E if only the first Oxford WebQ was considered (Online Resource 10a), minimum intake values were used (Online Resource 13a), and in males only (Online Resource 16a). Similar to the primary analysis, intrinsic sugars were not significantly associated with depression risk in all sensitivity analyses (Online Resource 5b–16b) except when minimum intake values were used (Online Resource 13b).

### FS in beverages versus FS in solids

Mean (SD) intake of FS in beverages and FS in solids was 4.9 (5.1) and 6.5 (3.5) %E, respectively (Table 1). Intake of FS in beverages was significantly associated with depression risk in an ascending approximately linear way (Fig. 1c). The HR-nadir for FS in beverages was observed at 4%E and the HRs (CIs) increased to 1.06 (1.02–1.11) and 1.31 (1.18–1.45) at 10%E and 20%E, respectively (Fig. 1c). The relation between FS in beverages and incident depression remained similar in all sensitivity analyses with the HR-nadir ranging from 0 to 7%E (Online Resource 5c–16c). FS in solids were not significantly related to depression risk in the primary (Fig. 1d) and in all sensitivity analyses (Online Resource 5d–16d).

### FS in beverage subtypes

**Fig. 2:**
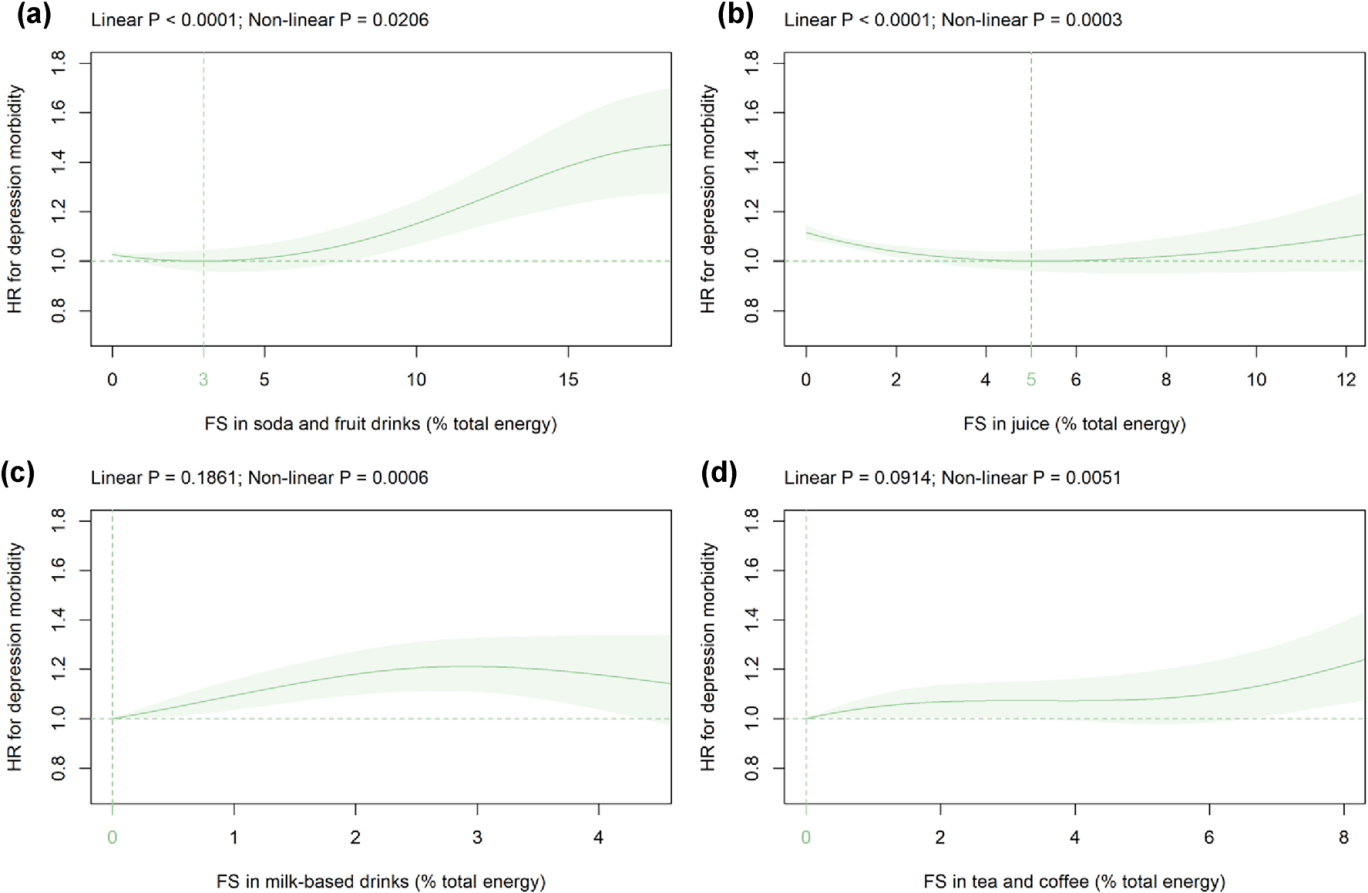
Association of FS in **a** soda/fruit drinks, **b** juice, **c** milk-based drinks, and **d** tea/coffee (all %E) with depression risk. Models are adjusted and presented as indicated in Fig. 1

Mean (SD) intake of FS in beverage subtypes was: soda/fruit drinks 1.8 (3.8), juice 2.1 (2.8), milk-based drinks 0.3 (0.9), and tea/coffee 0.6 (1.7) %E (Table 1). FS in soda/fruit drinks were significantly associated with depression risk in a linear fashion with the HR-nadir found at 3%E and HR (CI) of 1.15 (1.07–1.24) at 10%E (Fig. 2a). FS in juice were significantly associated with HR for depression in a U-shaped fashion with the HR-nadir observed at 5%E and HR (CI) of 1.12 (1.09–1.14) at 0%E (Fig. 2b). FS in milk-based drinks were significantly associated with depression risk in a non-linear wave-shaped fashion with the HR-nadir detected at 0%E and increased HR up to 3%E but not beyond this level (Fig. 2c). FS in tea/coffee were significantly related to incident depression in a curvilinear fashion with the HR-nadir detected at 0%E (Fig. 2d). These findings were robust in all sensitivity analyses with the following exceptions: the association between FS in milk-based drinks and tea/coffee did not remain statistically significant if only males were studied (Online Resource 16g, h).

### FS in solids subtypes

**Fig. 3:**
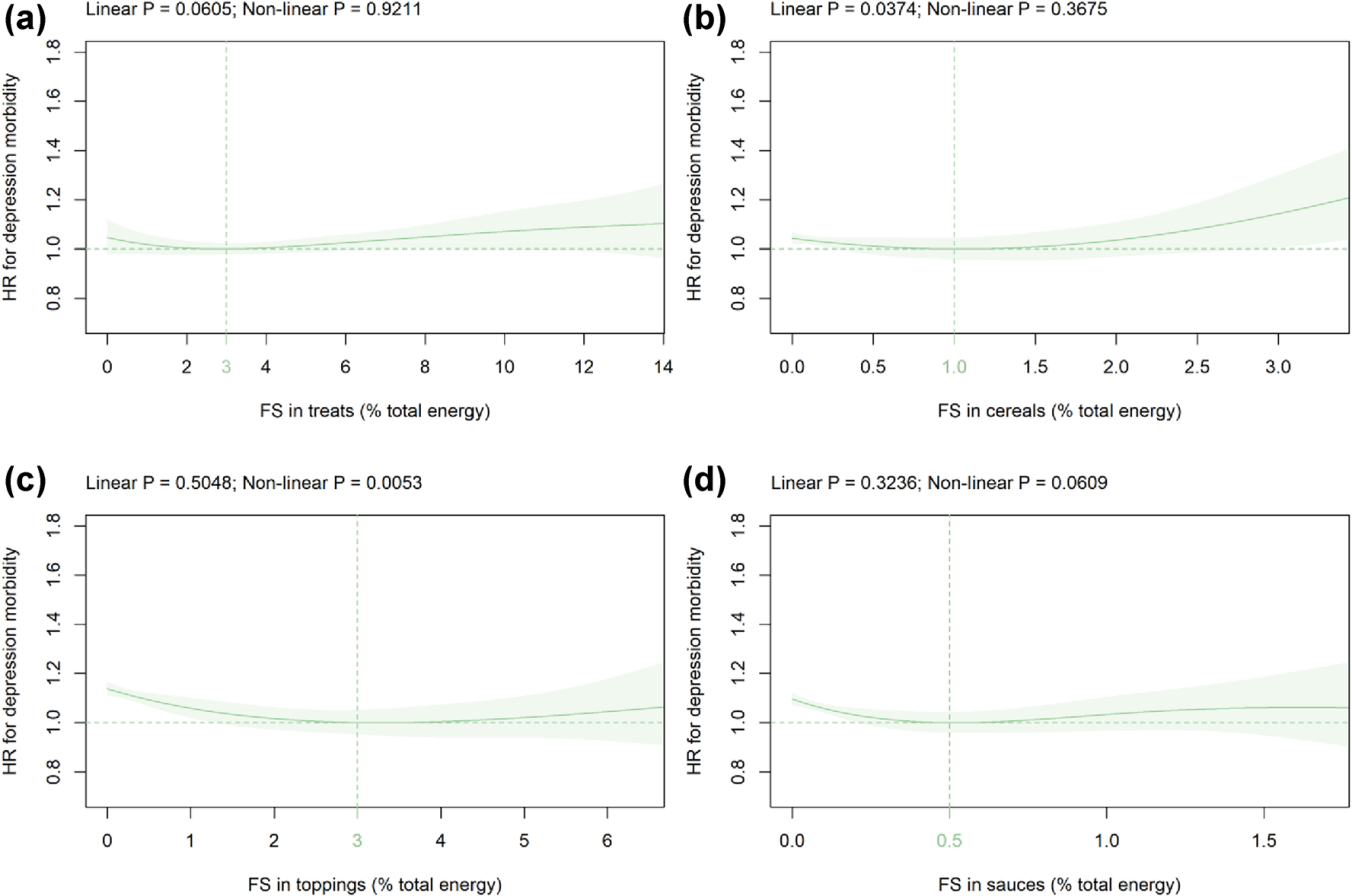
Association of FS in **a** treats, **b** cereals,** c** toppings, and **d** sauces (all %E) with depression risk. Models are adjusted and presented as indicated in Fig. 1

Mean (SD) intake of FS in solids subtypes was as follows: treats 4.3 (3.0), cereals 0.5 (0.8), toppings 1.2 (1.6), and sauces 0.3 (0.4) %E (Table 1). Within solids subtypes, FS in treats were not significantly related to depression risk in the primary cohort (Fig. 3a). A non-significant association was observed in all sensitivity analyses except when adjusting for a diet quality score (Online Resources 12i), considering minimum intake levels (Online Resource 13i), and females only (Online Resource 15i). FS in cereals were significantly related to incident depression in a linear manner in the primary analysis (Fig. 3b); however, the association remained statistically significant in only four out of the 12 sensitivity analyses (Online Resource 9j, 11j, 12j, and 16j). FS in toppings were significantly related to incident depression in a non-linear fashion in the primary analysis with the HR-nadir at 3%E and an increased HR in non-consumers (Fig. 3c). However, this association did not remain statistically significant in several sensitivity analyses (Online Resource 6k, 8k, 10k, 13k, and 16k). FS in sauces were not significantly related to depression risk in the primary analysis (Fig. 3d) and all sensitivity analyses except when varied sugar added to tea/coffee was removed (Online Resource 9l), considering maximum intake levels (Online Resource 14l), and males only (Online Resource 16l).

## Discussion

### Principal findings

The current study is the first to systematically assess the association of FS from all major sources with incident depression and some sugar sources are evaluated for the first time.

FS intake is significantly associated with depression risk in an ascending approximately linear way and a HR-nadir at 9%E. In contrast, intrinsic sugars are not significantly related to incident depression. FS in beverages are significantly associated with depression risk in an ascending approximately linear way whereas no association is found for FS in solids. Within beverages, FS in soda/fruit drinks, milk-based drinks, and tea/coffee are significantly and positively related to depression risk whereas the association is U-shaped for juice. In contrast, relations of FS in solids subtypes with incident depression are weaker and less robust in sensitivity analyses as compared to FS in beverages and beverage subtypes. Our results highlight that the associations between FS and depression risk depend on FS source.

### Comparison with other studies

Mean FS consumption of UK Biobank participants in the present study is comparable to representative data for the UK population from the National Diet and Nutrition Survey Rolling Programme 2014–2016 [39]. However, mean FS intake of the UK Biobank population is higher as recommended by the WHO [16] and the NHS [17]. In our study, FS consumption and depression risk are significantly associated in an ascending approximately linear way with the HR-nadir observed at 9%E. Similar to our present findings, Sánchez-Villegas et al. demonstrate convincingly that HR for depression is significantly increased 1.35-fold above the highest as compared to below the lowest quartile of added sugars consumption in 15,546 participants [18]. In contrast, no significant association between sugar intake from sweet food and beverages combined and incident depression 5 years later is observed in another report [40]. Differences in results might be well explained by different study sizes, as well as different proportions of FS subtypes in the respective studies.

To the best of our knowledge, the association of intrinsic sugars with depression risk is defined for the first time in the current study. In contrast to FS, intrinsic sugars are not significantly related to incident depression. Intrinsic sugars are preferentially found within the structure of intact fruit and vegetables or are naturally present as lactose and galactose in milk [16]. Interestingly, the pooled relative depression risk is reduced by 17% for fruit intake and by 14% for vegetable consumption in the highest versus the lowest category in a recent meta-analysis [41]. Therefore, the numerous health-promoting nutrients, e.g., vitamins, phytochemicals, and dietary fibres rather than intrinsic sugars might have a beneficial impact on incident depression. The current report supports the recommendation by the WHO that intrinsic sugars and FS should be distinguished since their physiological effects are different with FS but not intrinsic sugars being associated with adverse metabolic effects at higher consumption levels [16].

FS in beverages are significantly associated with depression in an ascending approximately linear way in the current report. To the best of our knowledge, no study of a prospective cohort so far has assessed the association of all FS in beverages combined with depression. Rather, reports have focused on soda/fruit drinks with some studies showing a positive association with depression [19–21] similar to our present findings while others present inconclusive evidence [18, 40]. Different results might be explained by differences in sample sizes, study cohorts, as well as definitions of depression and sugary beverages. It is important to note in this context that by far the two largest studies, i.e., the study by Guo et al. [19] (*n* = 263,923) and our present analysis (*n* = 188,426) show a dose-dependency of depression risk on soda/fruit drinks consumption. Our study is the first to elucidate the association between FS in milk-based drinks and juice on one hand and depression risk on the other hand. FS in milk-based drinks are positively associated with incident depression in a non-linear wave-shaped fashion. In contrast, the link between FS in juice and depression is rather U-shaped. It is interesting to note in this context that daily consumption of 380 ml of orange juice for 8 weeks significantly decreases depressive symptoms of young adults in a recent intervention study [42]. The consumption of about 30 g/day FS from juice derived from this juice intake are close to the HR-nadir of 5%E which is equivalent to about 26 g/day FS from juice in the present study. Combined, these data suggest that moderate juice intake might be a protective factor for depression. Only one study so far has evaluated the association between FS in tea/coffee and depression risk. Guo et al. demonstrate convincingly that depression risk of US adults who add sugar or honey to their tea or coffee is not significantly altered as compared to non-drinkers [19]. In the present study, the association between FS in tea/coffee and depression is curvilinear with the HR-nadir detected at 0%E. Together, these data suggest that the association between FS and depression depends on beverage type.

Our study is the first to analyse the association between FS in solids and depression risk. In contrast to FS in beverages, no link between FS in solids and depression is found. Furthermore, the relations of FS in solids subtypes with incident depression are weaker and less robust in sensitivity analyses as compared to FS in beverages and beverage subtypes. In agreement with our findings, no significant association between the consumption of commercial baked goods and depression is found in an independent report [43]. In contrast, the risk for depressive symptoms is significantly increased 1.7-fold above the upper tertile in comparison to below the lower tertile of confectionery consumption in another study [44]. Possible different physiological effects of FS from solids and beverages might be due to faster gastric emptying of beverages as compared to solids [45]. Indeed, high glycemic index diets are associated with an increased depression risk in cohort studies and clinical trials [46]. Together, these data suggest that FS from beverages and solids show distinct physiological effects and that FS from solids are not linked with depression risk. It is interesting to note in this context that significant differences concerning subjective feelings of hunger, fullness, and satiety can be observed between liquid and solid carbohydrate foods despite similar effects on glycemic and insulin responses [47].

Several mechanisms by which FS might cause depression have been proposed. Thus, a diet rich in saturated fat and refined sugar decreases BDNF in rats [22]. Decreased circulating BDNF has been linked to depression in humans and significantly higher BDNF levels are found after antidepressant treatment in a meta-analysis [48]. Furthermore, addiction-like effects have been described for sugar in rats with signs of behavioral depression and anxiety observed after sugar withdrawal [24]. These behaviors are related to changes in dopamine and opioid receptor binding, as well as dopamine and acetylcholine release in the nucleus accumbens [24]. Moreover, high glucose consumption might contribute to low-grade inflammation [23] which has been linked to the development of depression [49].

### Strengths and limitations of this study

Strengths of the current study include a large sample size, the prospective cohort design, thorough characterization of participants, mean follow-up > 12 years, a wide range of sugar subtype intake, as well as analyses with penalized cubic splines to allow non-linear predictor effects. Limitations include residual confounding, measurement errors in the assessment of the exposure variables, and potential confounders. Moreover, a “healthy volunteer” selection bias is possible since the cohort is not demographically representative of the general UK population [50]. However, a representative population is not required to define exposure–disease relationships [50]. Furthermore, dietary habits may not be constant over time and a dietary change may take time to impact depression. It remains to be elucidated how long this dietary change would have to be maintained to have a significant effect on mental health. In addition, some reverse causation cannot be excluded since a subclinical non-diagnosed depression might already have an impact on nutrition patterns including sugar subtype intake. However, results are not substantially altered in the landmark analysis. Moreover, all consumption data have not been independently assessed but self-reported. Since sugar has not been assessed in urine samples in UK Biobank participants, our current results cannot be confirmed with urinary sugar excretion as a biomarker of sugar intake [51].

### Conclusions and policy implications

Only some sources of FS are positively associated with incident depression. Public health initiatives targeting FS subtypes might be most effective concerning depression risk if focused on the reduction of sugary beverages and more specifically soda/fruit drinks, milk-based drinks, and tea/coffee. Further prospective studies on sugar subtype intake in relation to other disease states including cardiovascular disease and cancer are necessary to provide an even more definitive conclusion.

## Supplementary Information

Below is the link to the electronic supplementary material.

Supplementary file1 (PDF 4579 KB)

## Data Availability

Data supporting the results of this study are available from UK Biobank, but restrictions apply to the availability of these data, which were used under license for Application 53438, and so are not publicly available.
