# Supplementary material for: Association of sugar intake from different sources with incident depression in the prospective cohort of UK Biobank participants
Source: Eur J Nutr. 2022 Oct 7;62(2):727–38. doi: 10.1007/s00394-022-03022-7 (PMC9941260; doi:10.1007/s00394-022-03022-7)
Supplement: Supplementary file 1 — Supplementary file1 (PDF 4579 KB) [file 394_2022_3022_MOESM1_ESM.pdf]

Supplementary Material

European Journal of Nutrition

Association of sugar intake from different sources with incident depression in the prospective cohort of UK Biobank participants

Anna Kaiser[1#*]; Sylva M Schaefer[1#]; Inken Behrendt[1]; Gerrit Eichner[2§]; Mathias Fasshauer[1,3§]

1. Institute of Nutritional Science, Justus-Liebig University of Giessen, Giessen, Germany.
2. Mathematical Institute, Justus-Liebig University of Giessen, Giessen, Germany.
3. Department of Internal Medicine (Endocrinology, Nephrology, and Rheumatology), University of Leipzig, Leipzig, Germany.

[#]AK and SMS contributed equally to this work and are joint first authors.

[§]GE and MF contributed equally to this work and are joint senior authors.

*Corresponding author. Mailing address: Goethestr. 55, 35390 Giessen, Germany. Phone: +49 641 9939067. E-mail: anna.kaiser@ernaehrung.uni-giessen.de

Index

Online Resource 1

Sugar sources relevant to the present study

Online Resource 2

Flowchart of participant selection

Online Resource 3

Venn diagram depicting number of participants excluded by six exclusion criteria: 1) diagnosis of depression before completion of last Oxford WebQ, 2) missing socioeconomic factors (Townsend deprivation index, total household income, ethnic background, highest qualification, or overall health rating), 3) missing data of the physical exam (BMI or SBP), 4) being in the upper 0.1 % of total energy and/or carbohydrate intake or total energy intake of 0 kJ/d, 5) malabsorption, and 6) missing lifestyle risk factors (physical activity or smoking status) resulting in a study population of 188,426 participants.

Online Resource 4

Six-step procedure to estimate FS content

Online Resource 5

Landmark analysis

Association of **a** FS, **b** intrinsic sugars, as well as FS in **c** beverages, **d** solids, **e** soda/fruit drinks, **f** juice, **g** milk-based drinks, **h** tea/coffee, **i** treats, **j** cereals, **k** toppings, and **l** sauces (all %E) with depression risk (landmark analysis; n=187,943; number of cases=5,215). Models are adjusted for energy intake, age, alcohol intake, BMI, ethnic background, general health status, history of mental illnesses, physical activity, SBP, sex, smoking status, total household income, and Townsend deprivation index as summarized in the Methods section. Covariates not fulfilling the proportional hazard assumption are stratified. The nadir is indicated in green. FS, Free sugars; HR, Hazard ratio

Online Resource 6

Unintentional weight loss removed

Association of **a** FS, **b** intrinsic sugars, as well as FS in **c** beverages, **d** solids, **e** soda/fruit drinks, **f** juice, **g** milk-based drinks, **h** tea/coffee, **i** treats, **j** cereals, **k** toppings, and **l** sauces (all %E) with depression risk (unintentional weight loss removed; n=157,633; number of cases=4,274). Models are adjusted and presented as indicated in supplementary Online Resource 5. FS, Free sugars; HR, Hazard ratio

Online Resource 7

Implausible energy intake removed

Association of **a** FS, **b** intrinsic sugars, as well as FS in **c** beverages, **d** solids, **e** soda/fruit drinks, **f** juice, **g** milk-based drinks, **h** tea/coffee, **i** treats, **j** cereals, **k** toppings, and **l** sauces (all %E) with depression risk (implausible energy intake removed; n=179,122; number of cases=5,022). Models are adjusted and presented as indicated in supplementary Online Resource 5. FS, Free sugars; HR, Hazard ratio

Online Resource 8

Non-typical diet removed

Association of **a** FS, **b** intrinsic sugars, as well as FS in **c** beverages, **d** solids, **e** soda/fruit drinks, **f** juice, **g** milk-based drinks, **h** tea/coffee, **i** treats, **j** cereals, **k** toppings, and **l** sauces (all %E) with depression risk (non-typical diet removed; n=127,894; number of cases=3,763). Models are adjusted and presented as indicated in supplementary Online Resource 5. FS, Free sugars; HR, Hazard ratio

Online Resource 9

"Varied" sugar added to tea/coffee removed

Association of **a** FS, **b** intrinsic sugars, as well as FS in **c** beverages, **d** solids, **e** soda/fruit drinks, **f** juice, **g** milk-based drinks, **h** tea/coffee, **i** treats, **j** cereals, **k** toppings, and **l** sauces (all %E) with depression risk ("Varied" sugar added to tea/coffee removed; n=187,422; number of cases=5,376). Models are adjusted and presented as indicated in supplementary Online Resource 5. FS, Free sugars; HR, Hazard ratio

Online Resource 10

First Oxford WebQ only

Association of **a** FS, **b** intrinsic sugars, as well as FS in **c** beverages, **d** solids, **e** soda/fruit drinks, **f** juice, **g** milk-based drinks, **h** tea/coffee, **i** treats, **j** cereals, **k** toppings, and **l** sauces (all %E) with depression risk. Only the first Oxford WebQ was used for intake estimation (n=188,426; number of cases=5,410). Models are adjusted and presented as indicated in supplementary Online Resource 5. FS, Free sugars; HR, Hazard ratio

Online Resource 11

Adjustment for WHR and height instead of BMI

Association of **a** FS, **b** intrinsic sugars, as well as FS in **c** beverages, **d** solids, **e** soda/fruit drinks, **f** juice, **g** milk-based drinks, **h** tea/coffee, **i** treats, **j** cereals, **k** toppings, and **l** sauces (all %E) with depression risk. Models were adjusted for WHR and height instead of BMI (n=188,382; number of cases=5,407). Models are adjusted and presented as indicated in supplementary Online Resource 5. FS, Free sugars; HR, Hazard ratio

Online Resource 12

Adjustment for diet quality score

Association of **a** FS, **b** intrinsic sugars, as well as FS in **c** beverages, **d** solids, **e** soda/fruit drinks, **f** juice, **g** milk-based drinks, **h** tea/coffee, **i** treats, **j** cereals, **k** toppings, and **l** sauces (all %E) with depression risk. Models were further adjusted for diet quality score (n=185,851; number of cases=5,280). Models are adjusted and presented as indicated in supplementary Online Resource 5. FS, Free sugars; HR, Hazard ratio

Online Resource 13

Minimum intake values

Association of **a** FS, **b** intrinsic sugars, as well as FS in **c** beverages, **d** solids, **e** soda/fruit drinks, **f** juice, **g** milk-based drinks, **h** tea/coffee, **i** treats, **j** cereals, **k** toppings, and **l** sauces (all %E) with depression risk in female participants only (n= 188,426; number of cases= 5,410). Models are adjusted and presented as indicated in supplementary Online Resource 5. FS, Free sugars; HR, Hazard ratio

Online Resource 14

Maximum intake values

Association of **a** FS, **b** intrinsic sugars, as well as FS in **c** beverages, **d** solids, **e** soda/fruit drinks, **f** juice, **g** milk-based drinks, **h** tea/coffee, **i** treats, **j** cereals, **k** toppings, and **l** sauces (all %E) with depression risk in male participants only (n= 188,426; number of cases= 5,410). Models are adjusted and presented as indicated in supplementary Online Resource 5. FS, Free sugars; HR, Hazard ratio

Online Resource 15

Female participants only

Association of **a** FS, **b** intrinsic sugars, as well as FS in **c** beverages, **d** solids, **e** soda/fruit drinks, **f** juice, **g** milk-based drinks, **h** tea/coffee, **i** treats, **j** cereals, **k** toppings, and **l** sauces (all %E) with depression risk in female participants only (n= 102,575; number of cases= 3,447). Models are adjusted and presented as indicated in supplementary Online Resource 5 except for sex. FS, Free sugars; HR, Hazard ratio

Online Resource 16

Male participants only

Association of **a** FS, **b** intrinsic sugars, as well as FS in **c** beverages, **d** solids, **e** soda/fruit drinks, **f** juice, **g** milk-based drinks, **h** tea/coffee, **i** treats, **j** cereals, **k** toppings, and **l** sauces (all %E) with depression risk in male participants only (n= 85,851; number of cases= 1,963). Models are adjusted and presented as indicated in supplementary Online Resource 5 except for sex. FS, Free sugars; HR, Hazard ratio

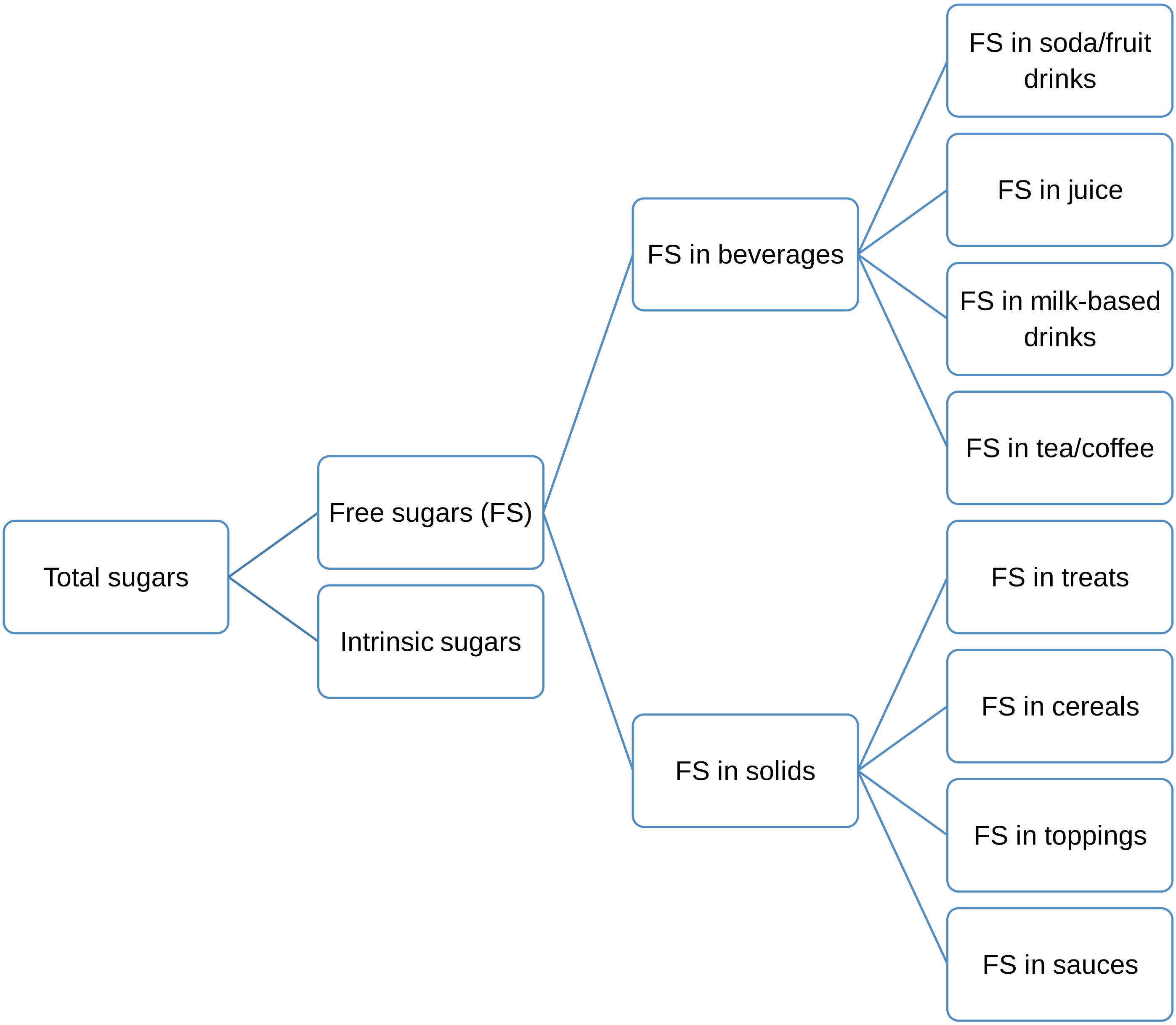

Online Resource 1

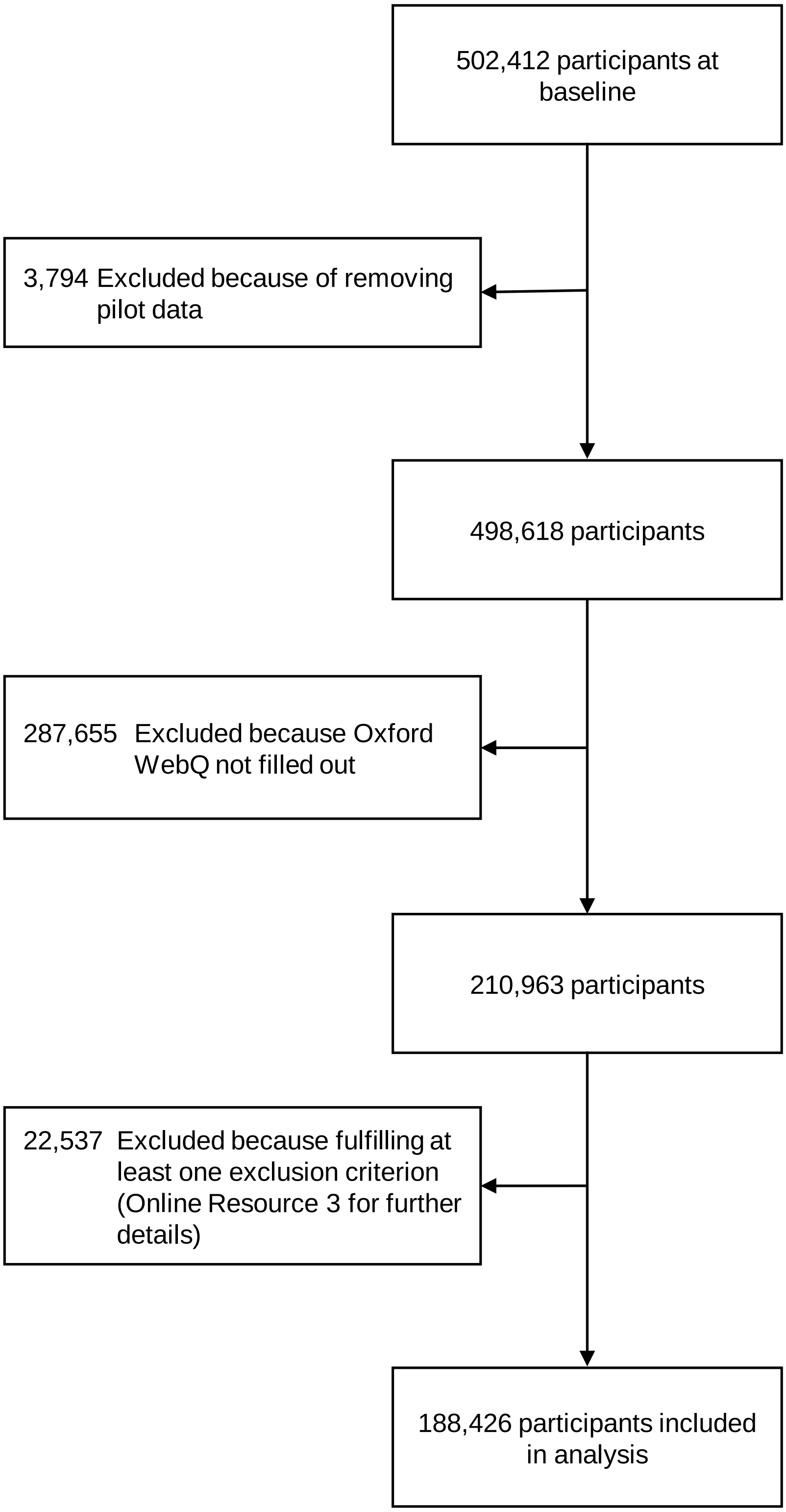

Online Resource 2

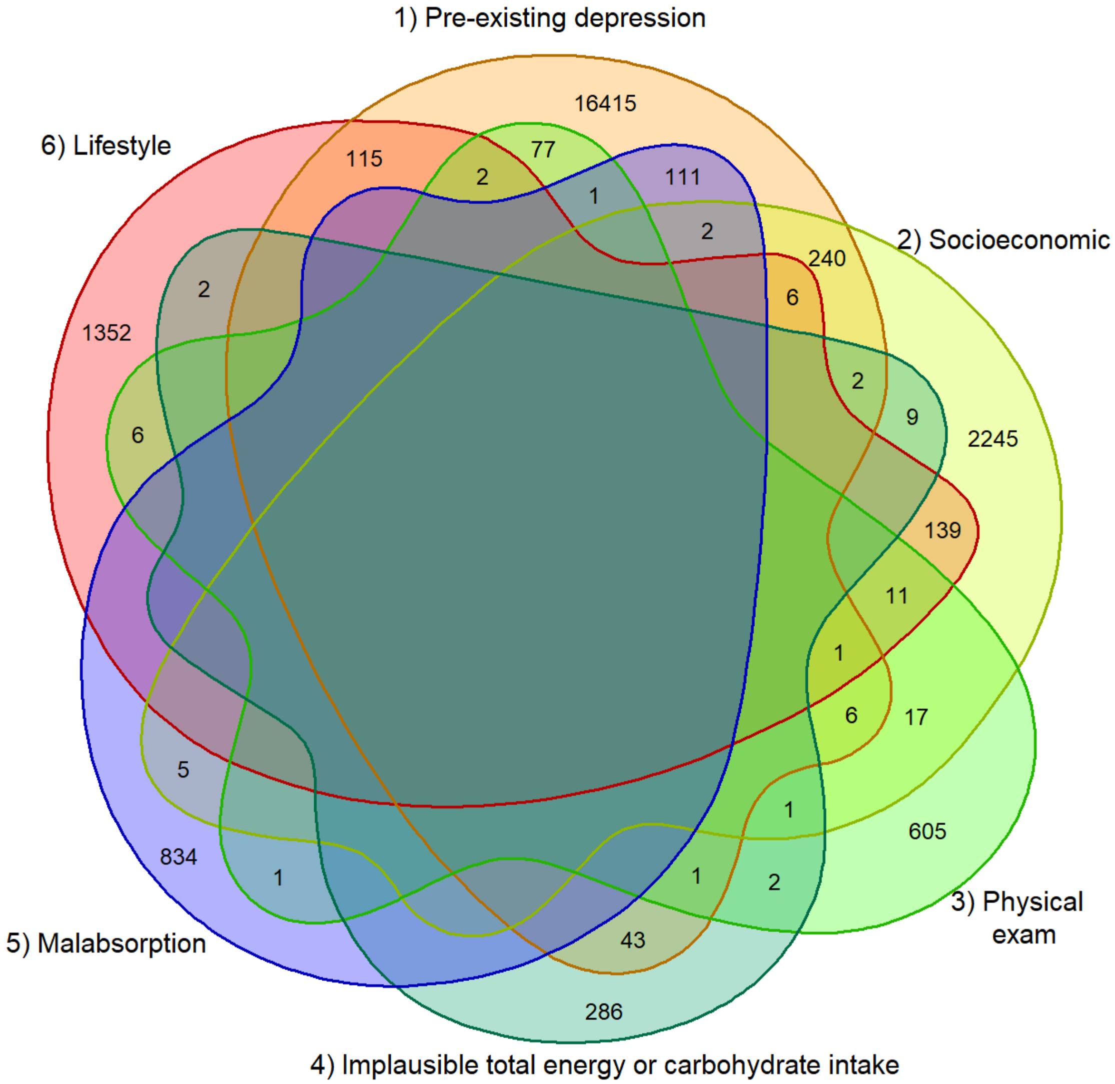

Online Resource 3

Online Resource 4 Six-step procedure to estimate free sugar (FS) content.[1]

**Step 1**	Food items containing 0 g of total sugars are assigned 0 g of FS.
**Step 2**	Food items in the food groups stated below are unprocessed or minimally processed with no FS and are assigned 0 g of FS:
	(a) spices and herbs<br>(b) fats and oils<br>(c) plain cereal grains, pseudocereals (e.g., buckwheat, quinoa, amaranth), flour, pasta, rice, plain cereal products, unsweetened potato chips<br>(d) plain breads with minimal amounts of FS only used for activation of yeast in fermentation (<9 g/>1000 g pre-baking weight)<br>(e) eggs and egg products (excluding egg-based desserts)<br>(f) fresh, frozen, or cooked fruits, berries, vegetables (including salads without dressing and root vegetables), unsweetened dried fruits<br>(g) fruit and vegetables canned in 100% vegetable juice or in artificially sweetened liquid<br>(h) unsweetened nuts, seeds, coconut, coconut products<br>(i) fresh meat, fresh fish, fresh seafood, tofu, unsweetened legumes, mushrooms, mixed meat dishes without FS<br>(j) coffee, tea, alcoholic beverages unsweetened or artificially sweetened<br>(k) unsweetened milk, unsweetened dairy products, non-dairy milk substitutes (e.g., oat and soy drinks and yoghurt)<br>(l) 100% vegetable juices; vegetable drinks sweetened with artificial sweeteners only<br>(m) jams, beverage bases, fruit curds or sauces that are unsweetened or artificially sweetened

**Step 3**	Food items in the food groups stated below are considered having minimal amounts of intrinsic sugars and are assigned 100% of total sugars as FS (total sugar values were estimated based on McCance and Widdowson's The Composition of Foods [1] and its supplements [2–10]):
	(a) confectionary except confectionary with dairy (e.g., chocolate, fudge)<br>(b) flavoured potato chips and other salty snacks<br>(c) breakfast cereals and bars (e.g., muesli bars) without fruits, chocolate, or dairy<br>(d) sugar-sweetened coffee and tea, beverage and soup bases with FS and without dairy (e.g., cordial)<br>(e) processed meats, fish, shellfish, blood products, and vegetarian dishes including pies, filled pastries, and breaded meats<br>(f) sugar-sweetened soda, sports drinks, flavoured water, and energy drinks without fruits<br>(g) baked food items such as cookies, buns, donuts, sponge cake, and other batter-based products without fruits, chocolate, or dairy<br>(h) plain pastries without fillings, fruits, dairy, nuts, or chocolate<br>(i) sugar-sweetened breads without fruits or dairy<br>(j) sugar-sweetened non-dairy milk substitutes (e.g., oat and soy drinks and yoghurt) without fruits<br>(k) table sugar, honey, and syrups<br>(l) sugar-sweetened non-dairy alcoholic beverages (e.g., punch)<br>(m) food items with FS that are pickled, cured, or marinated (e.g., pickled vegetables)<br>(n) sauces, dressings, and mayonnaise that contain FS<br>(o) fruit juice, fruit juice concentrate
**Step 4**	For food items not covered by steps 1 to 3, with analytical information on lactose available from [1–10], and not containing any fruits or vegetables, FS are calculated as sum of total sugars minus lactose.

**Step 5**	For composite food items not already covered by steps 1 to 4 and with total sugars content available from [1–10], FS content is calculated as follows:
	[1] Recipes are taken from the UKDA recipe database [11] or from McCance and Widdowson [1–10]<br>[2] FS and total sugars for individual recipe items are defined by steps 1 to 4<br>[3] The amount of FS and total sugars of each individual recipe item is multiplied with the percentage of this item within the recipe to calculate its content in 100 g recipe<br>[4] The individual recipe items are summed up to calculate FS and total sugar content in the composite food item<br>[5] The proportion of FS to total sugars is determined<br>[6] This proportion is multiplied with total sugars content from [1–10]
**Step 6**	For composite food items not already covered by steps 1 to 5 and with total sugars content **not** available from [1–10], FS content is calculated as follows:
	[1] Recipes are taken from the UKDA recipe database [11] or from McCance and Widdowson [1–10]<br>[2] FS for individual recipe items are defined by steps 1 to 4<br>[3] The amount of FS of each individual recipe item is multiplied with the percentage of this item within the recipe to calculate its content in 100 g recipe<br>[4] The individual recipe items are summed up to calculate FS content in the composite food item

[1]FS are all monosaccharides and disaccharides added to foods by the manufacturer, cook, or consumer, plus sugars naturally present in honey, syrups, and fruit juices.

References

1. McCance RA, Widdowson EM (2002) McCance and Widdowson's: The Composition of Foods: sixth summary edition, 06, 6th Summary edition. Royal Society of Chemistry, Cambridge
2. Holland B, Welch A, Buss D (1992, repr. 1996) Vegetable dishes: Second Supplement to McCance and Widdowson's the composition of Foods, 5th ed. Royal Society of Chemistry, Cambridge
3. Holland B, Unwin I, Buss D (1992) Fruit and Nuts: First Supplement to the Fifth Edition of McCance and Widdowson's The Composition of Foods, 5th ed. RSC Publishing, Cambridge
4. Holland B, Unwin I d., Buss DH et al. (1989) Milk Products and Eggs: Fourth Supplement to McCance and Widdowson's the Composition of Foods, 4th ed., 4. ed. The composition of foods, Supplement / Robert Alexander MacCance; Elsie M. Widdowson Hrsg. The Royal Society of Chemistry ; 4. Royal Society of Chemistry; Ministry of Agriculture Fisheries and Food, Cambridge, UK, London
5. Holland B, Unwin I, Buss D (eds) (1991) Vegetable, Herbs and Spices: Fifth Supplement to McCance and Widdowson's the Composition of Foods, 5th ed. Royal Society of Chemistry; Ministry of Agriculture Fisheries and Food, Cambridge, UK, London
6. Holland B, Brown J, Buss DH et al. (eds) (1993) Fish and Fish products.: Third supplement to McCance and Widdowson's the Composition of Food, 5th ed. Royal Society of Chemistry; Ministry of Agriculture Fisheries and Food, Cambridge, UK, London
7. Chan W, Bron J, Church S et al. (1996) Meat products and Dishes: Sixth Supplement to McCance and Widdowson's The composition of Foods, 5th ed. Royal Society of Chemistry and Ministry of Agriculture Fisheries and Food, Cambridge
8. Chan W, Brown J, Lee S et al. (1995) Meat poultry and Game: Fifth supplement to McCance and Widdowson's The composition of Foods, 5th ed. Royal Society of Chemistry; Ministry of Agriculture Fisheries and Food, Cambridge, London
9. Chan W, Buss DH, Brown J (1994) Miscellaneous Foods: Fourth supplement to McCance and Widdowson's the composition of Foods, 5th ed. Royal Society of Chemistry, Cambridge
10. McCance RA, Widdowson EM (2014) McCance and Widdowson's: The Composition of Foods: seventh summary edition. Royal Society of Chemistry
11. MRC Human Nutrition Research (2017) Food Standards Agency Standard Recipes Database, 1992-2012. [data collection]. UK Data Service. SN: 8159. https://doi.org/10.5255/UKDA-SN-8159-1

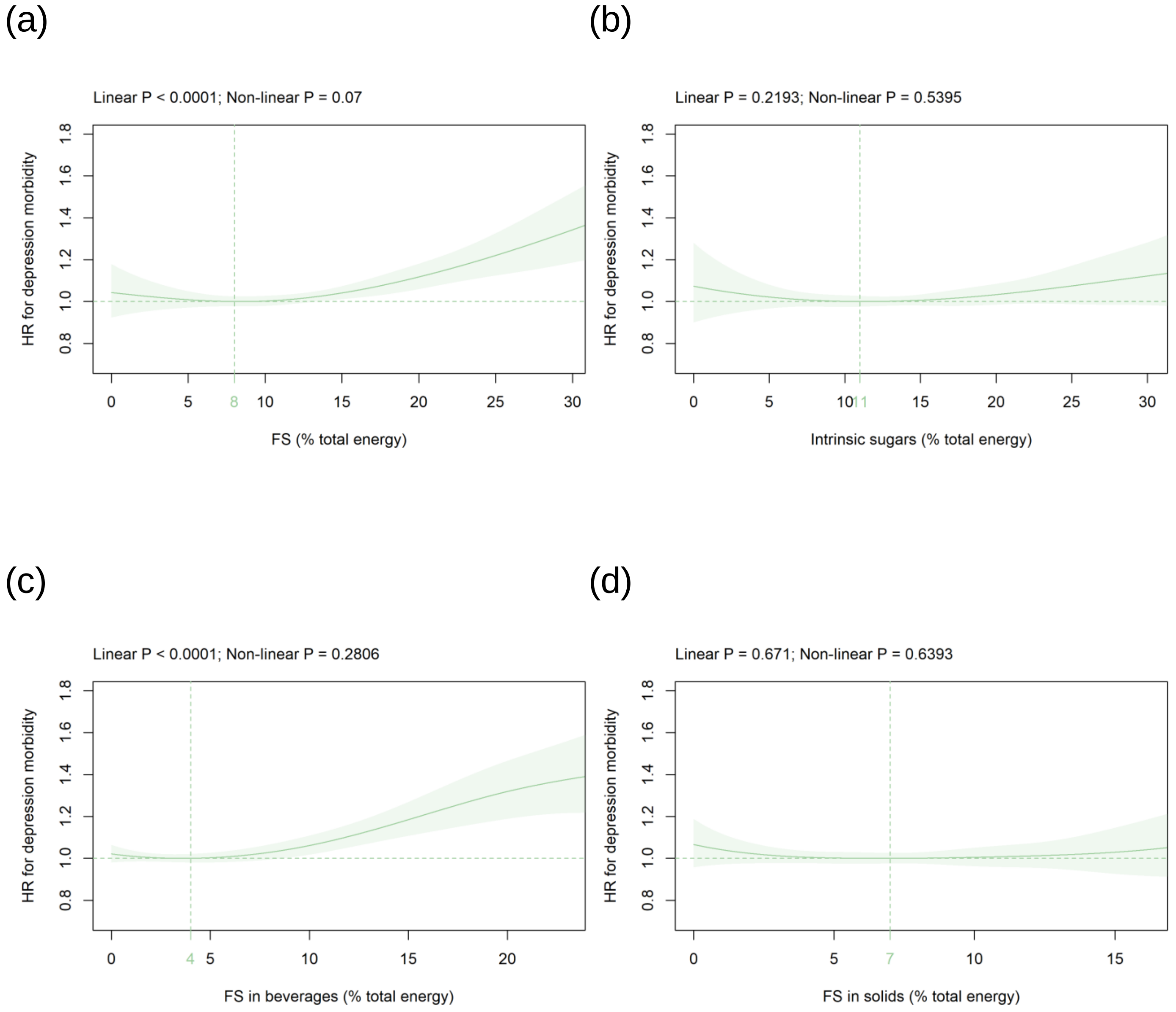

Online Resource 5

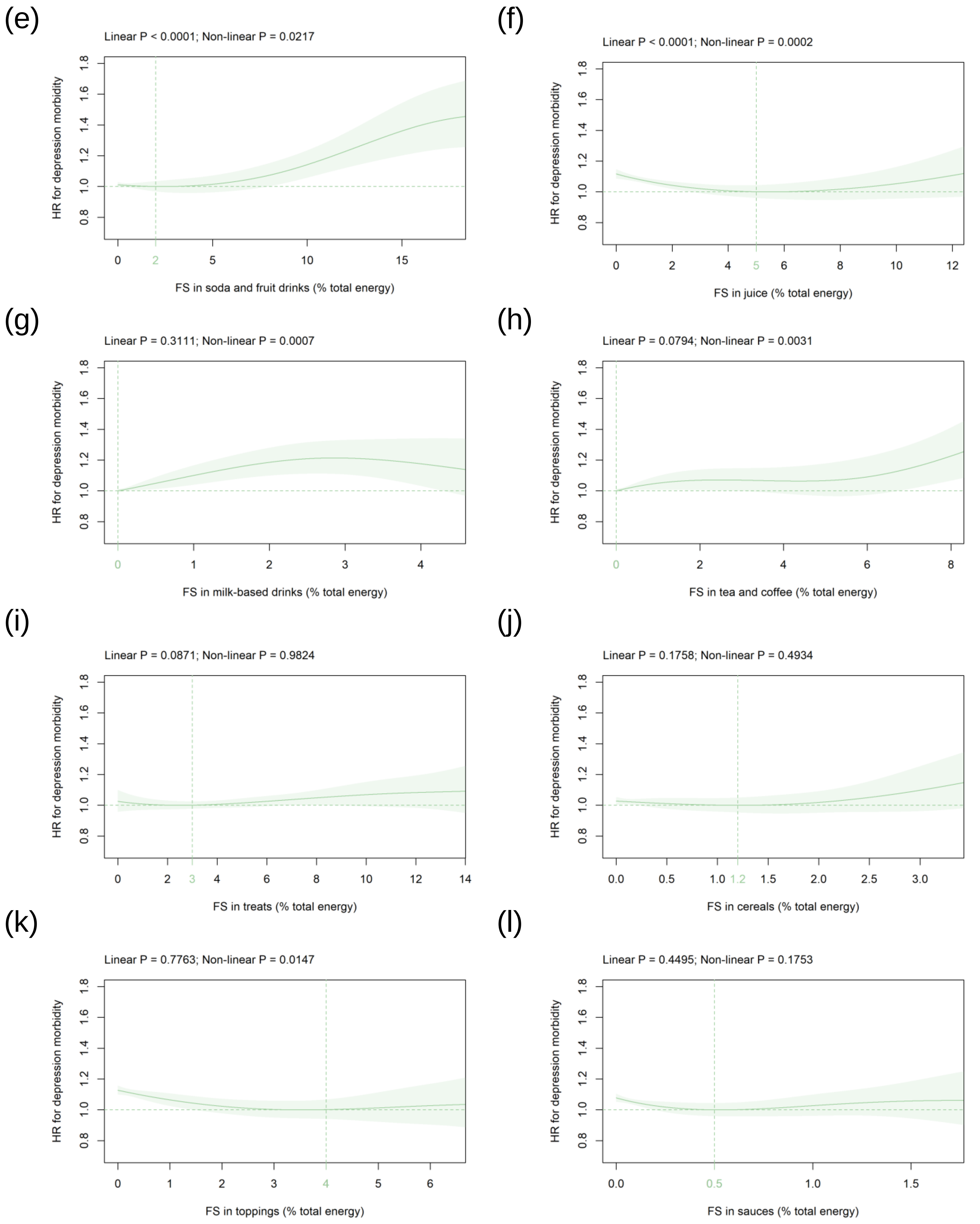

Online Resource 5 continued

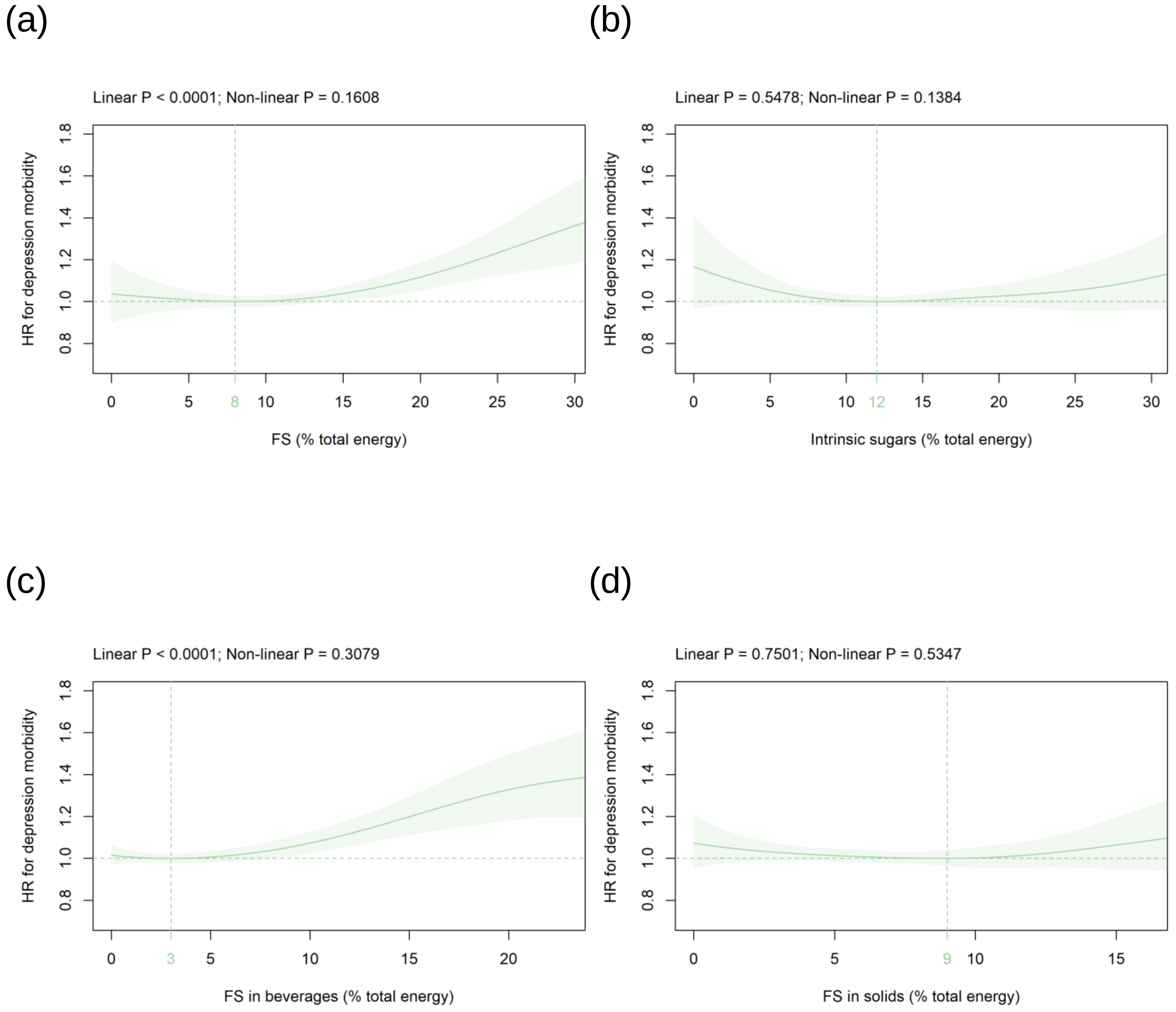

Online Resource 6

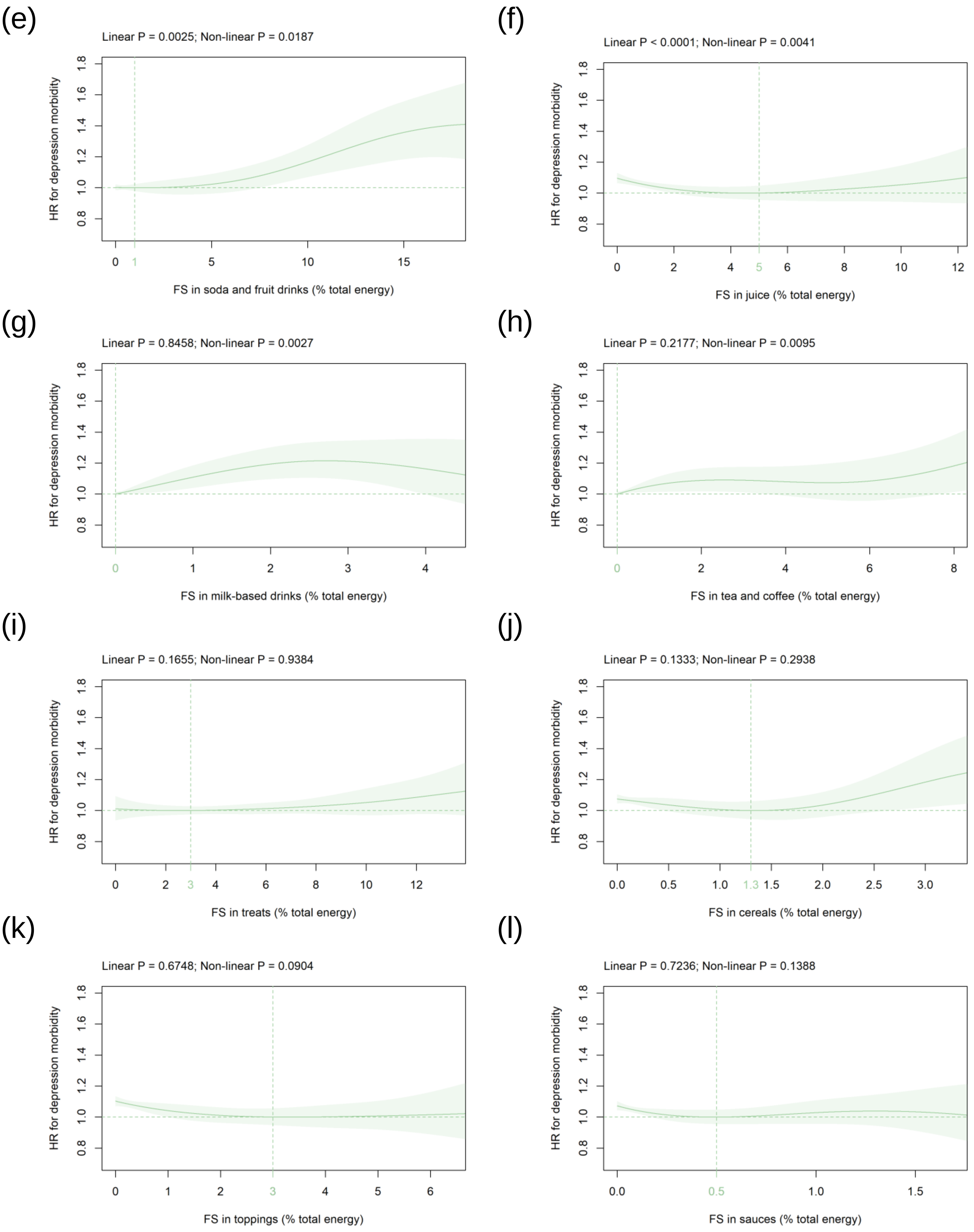

Online Resource 6 continued

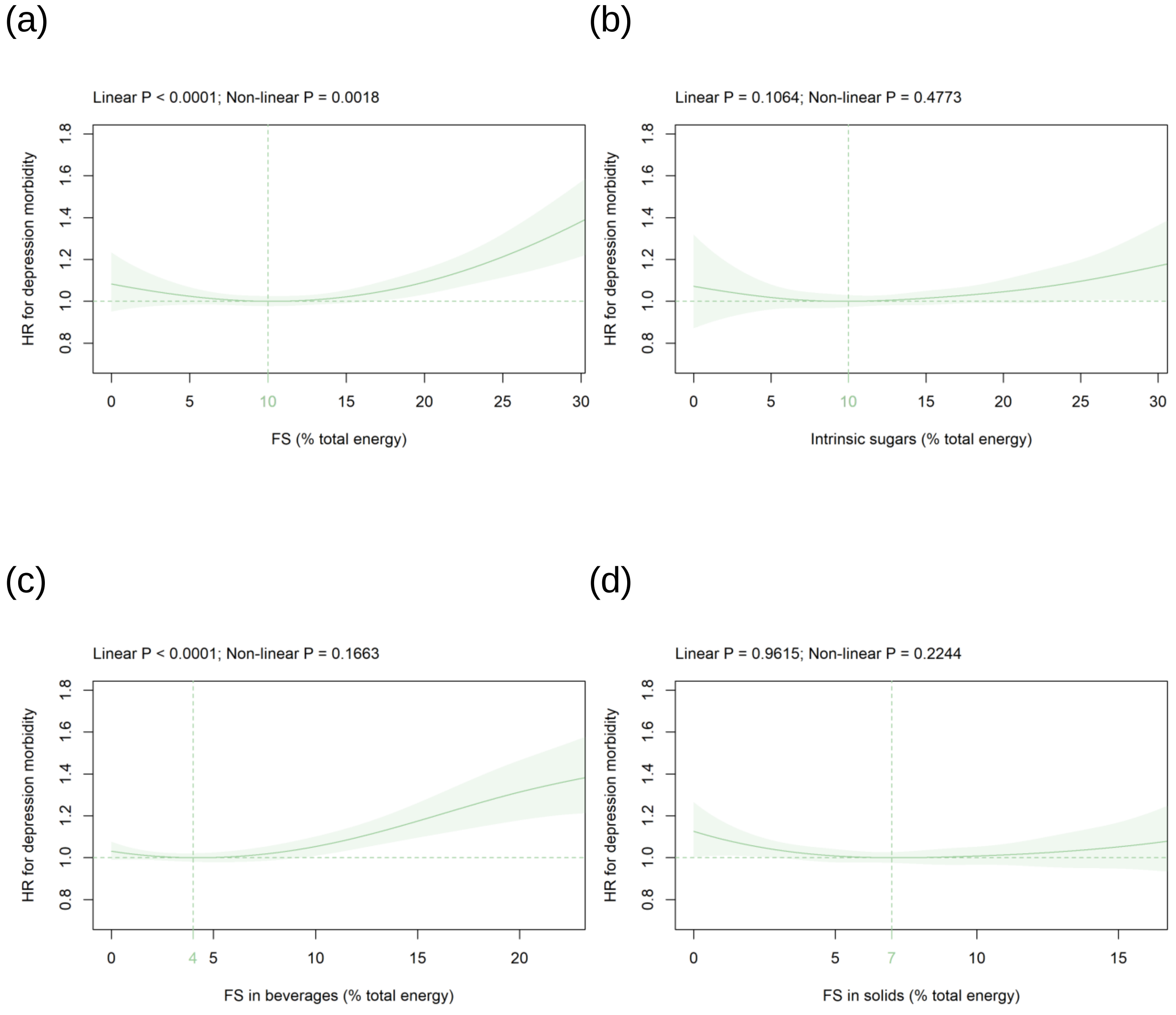

Online Resource 7

(e)

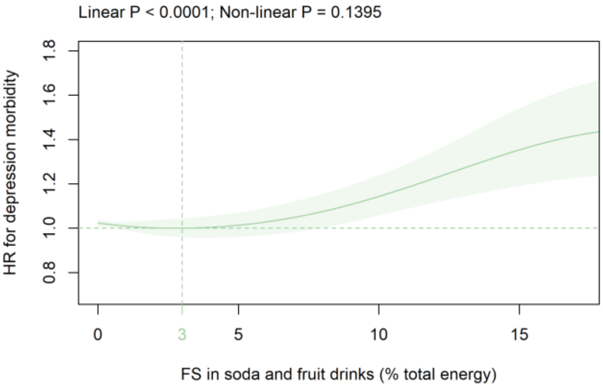

(f)

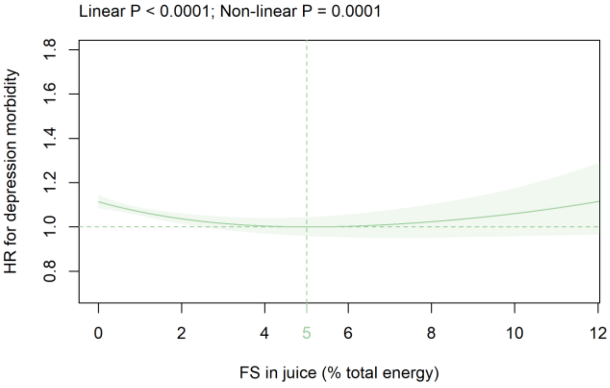

(g)

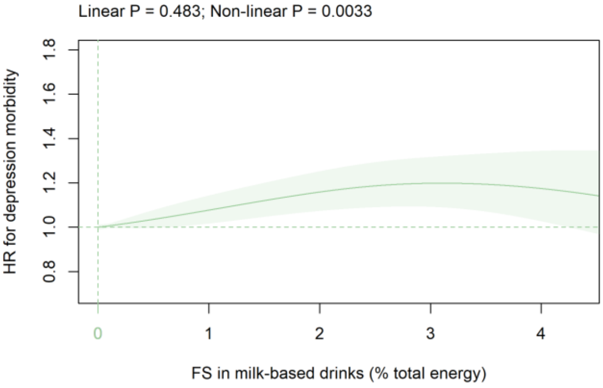

(h)

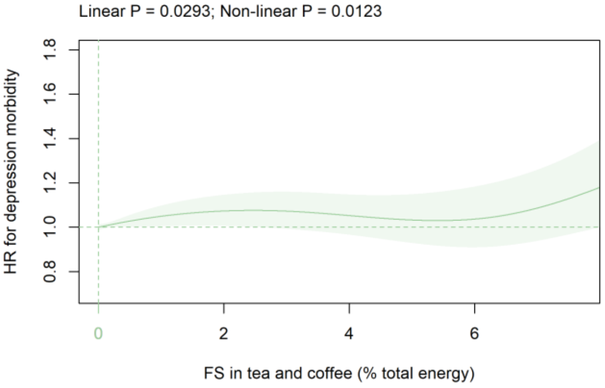

(i)

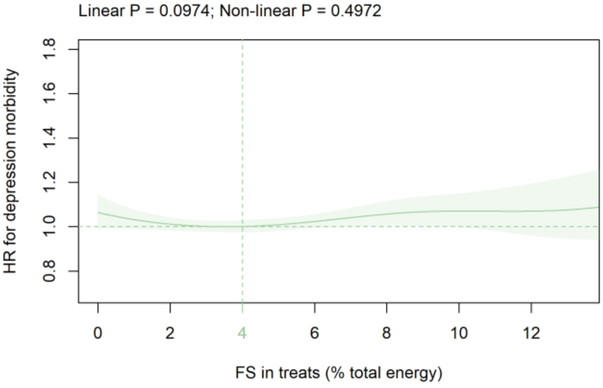

(j)

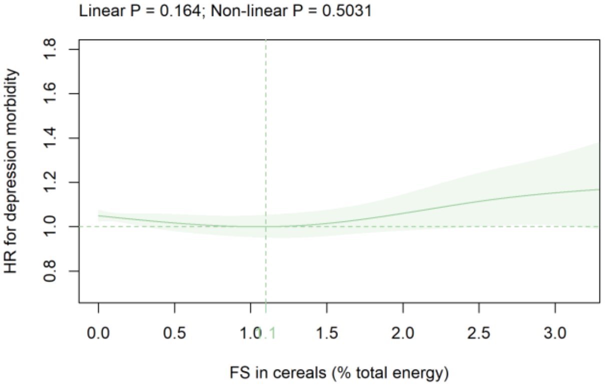

(k)

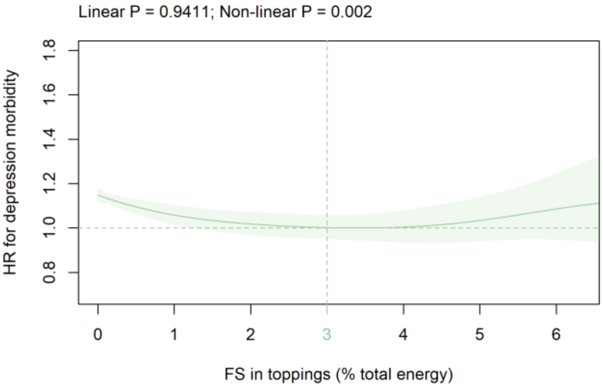

(l)

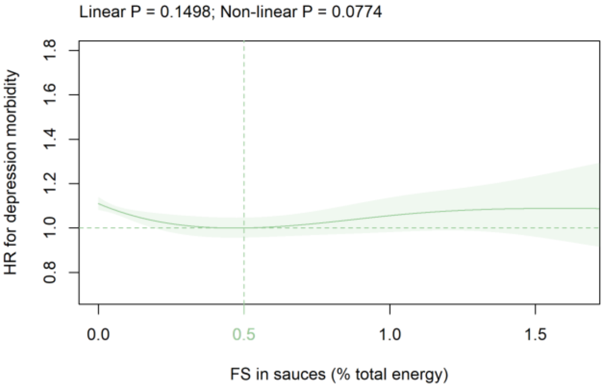

Online Resource 7 continued

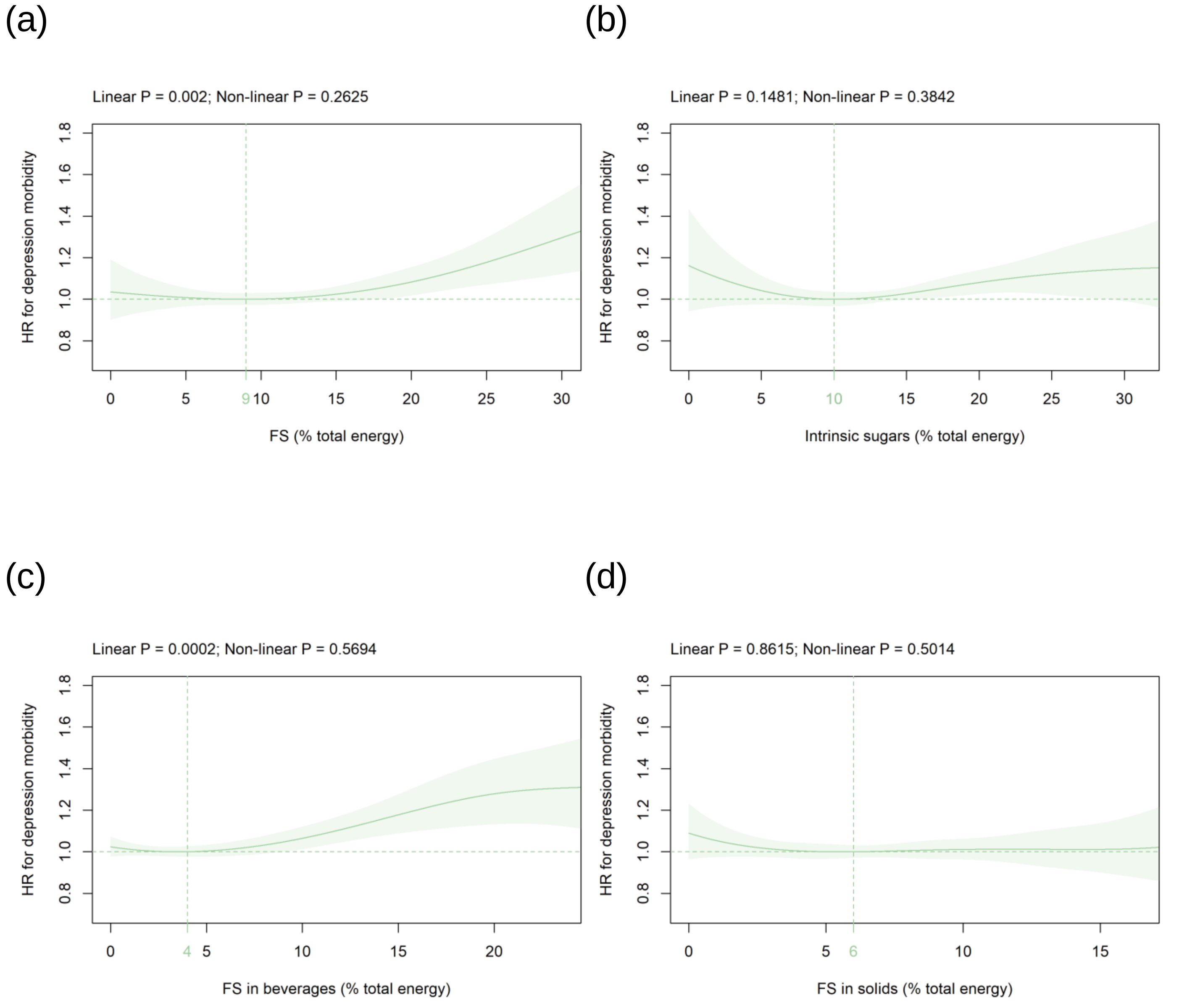

Online Resource 8

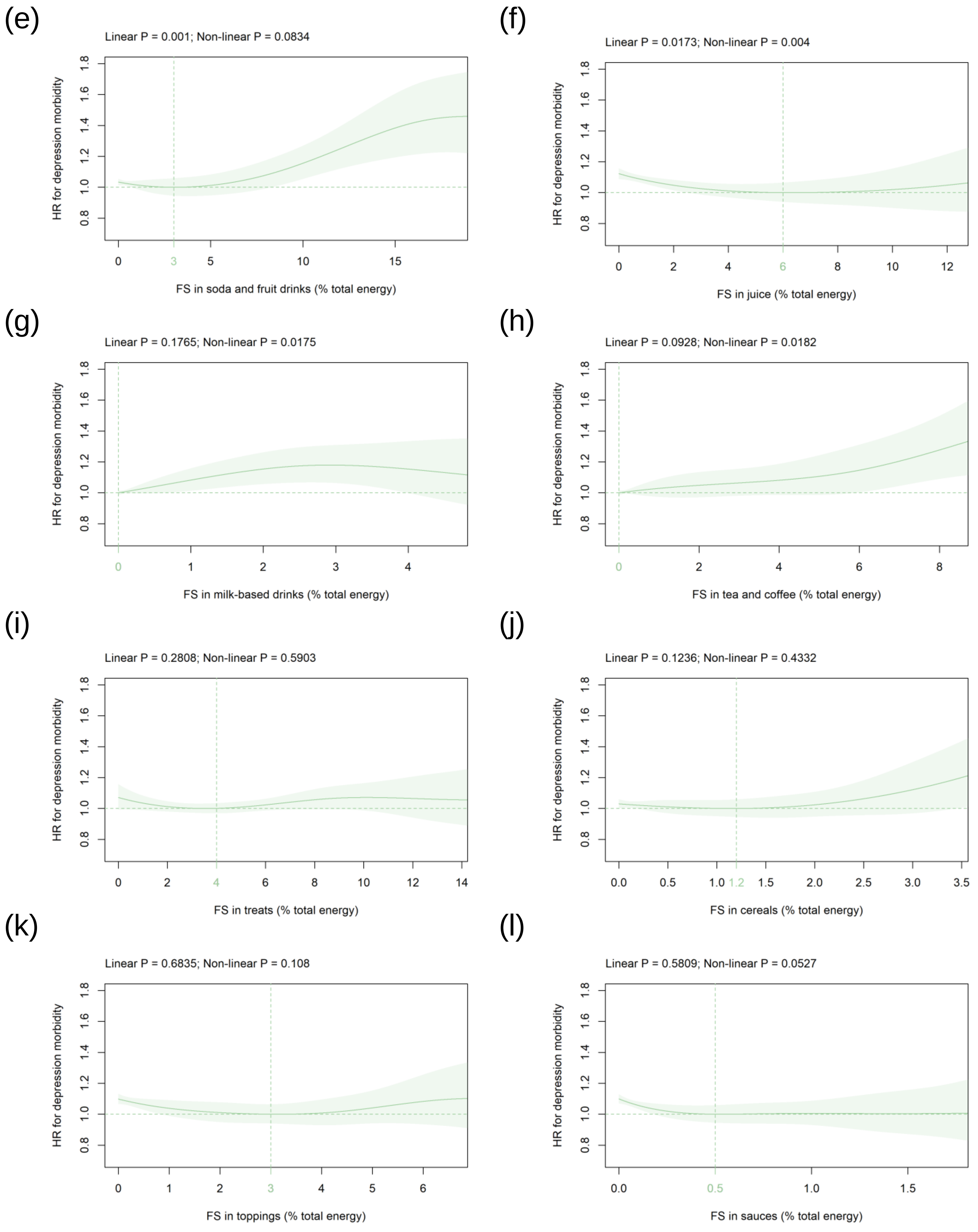

Online Resource 8 continued

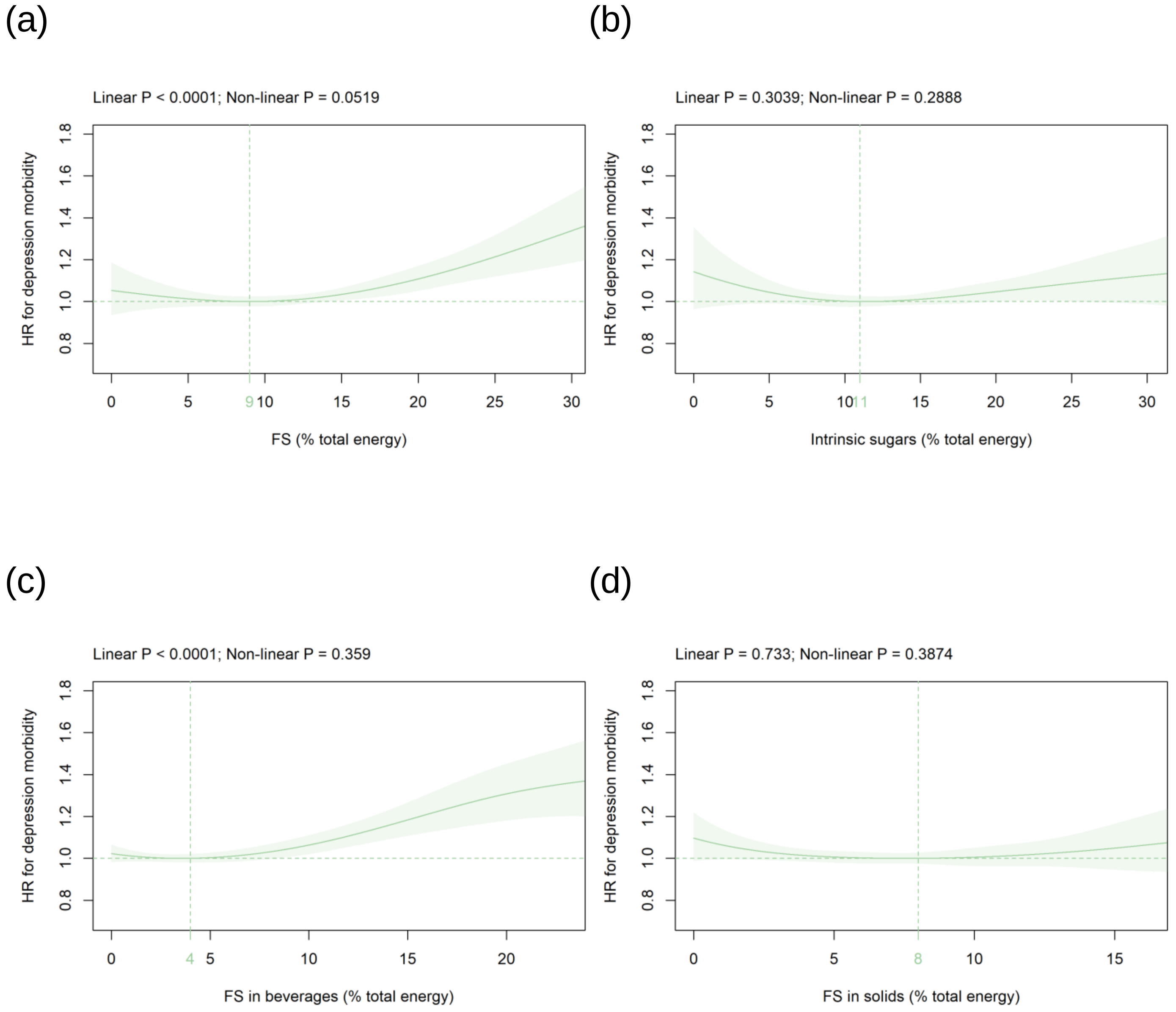

Online Resource 9

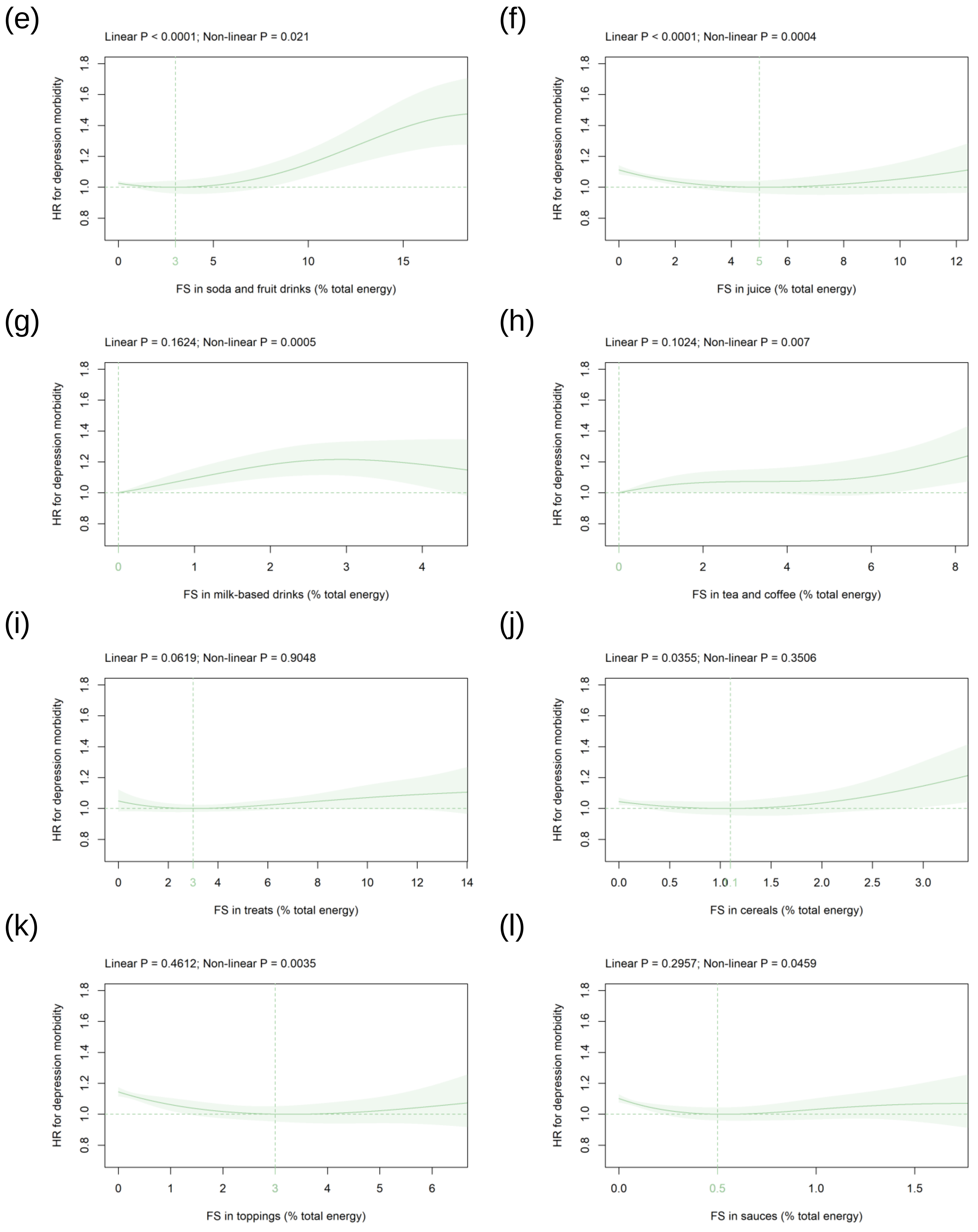

Online Resource 9 continued

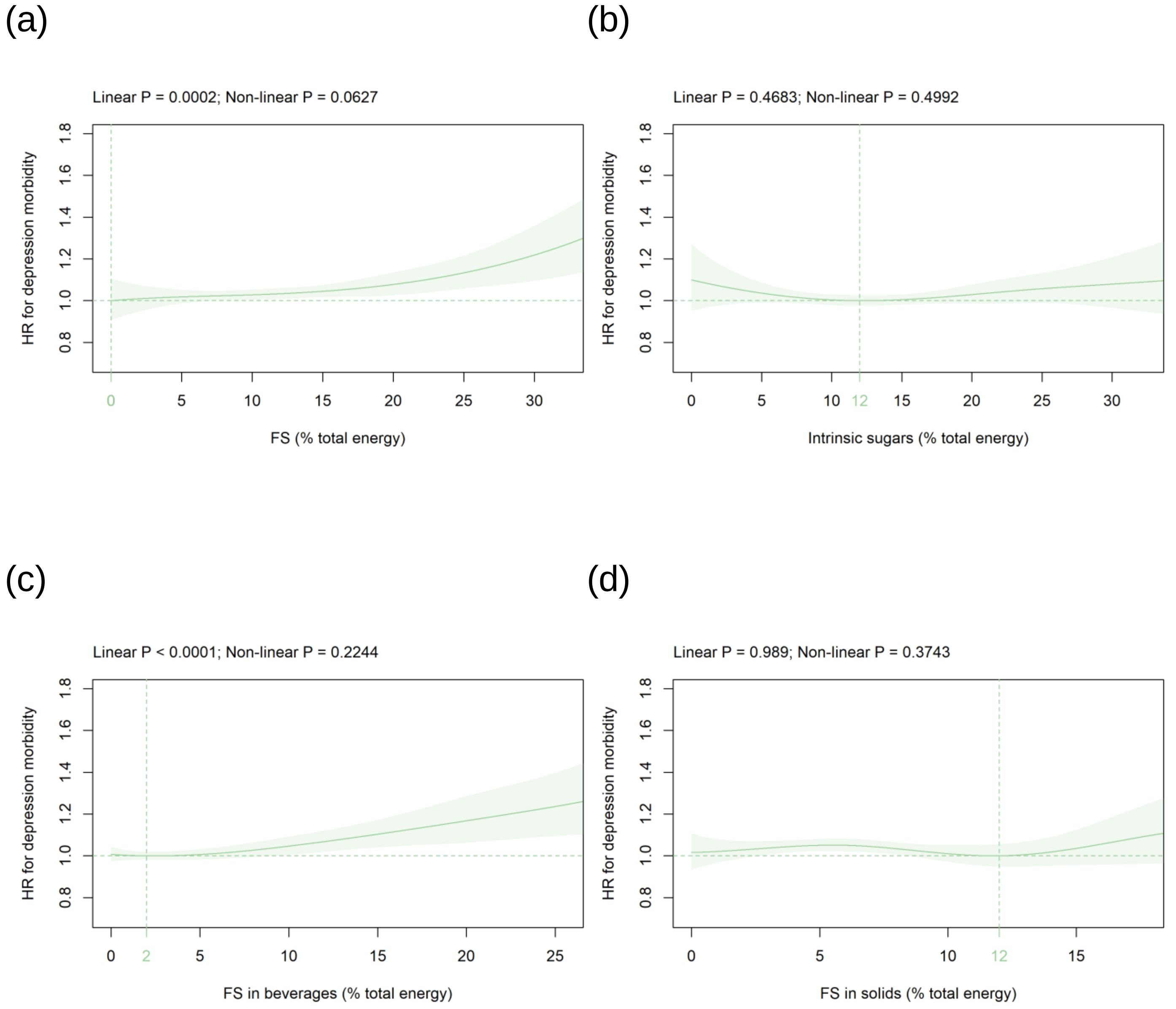

Online Resource 10

(e)

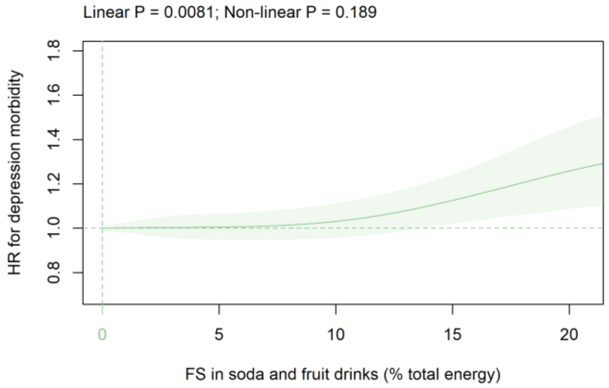

(f)

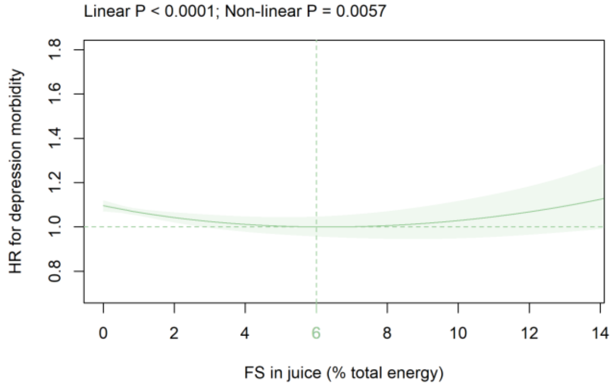

(g)

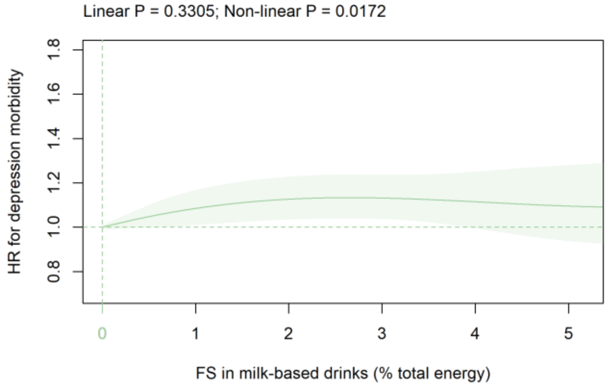

(h)

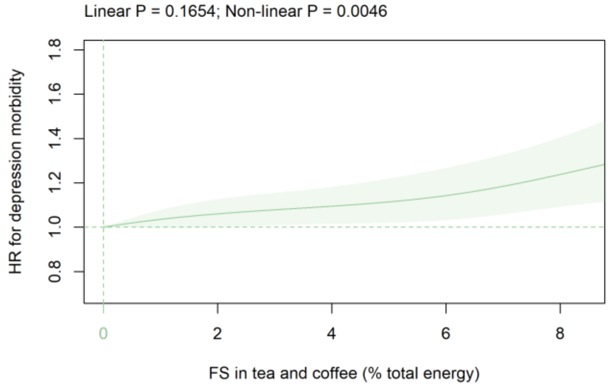

(i)

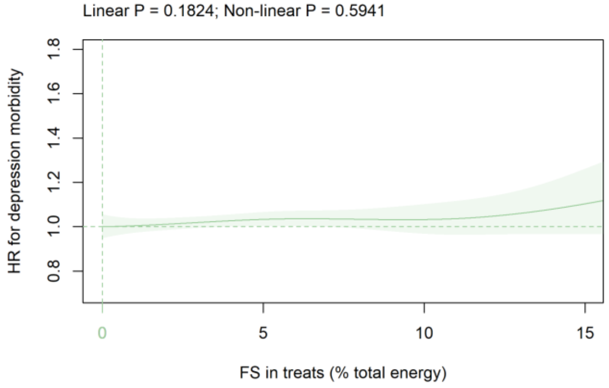

(j)

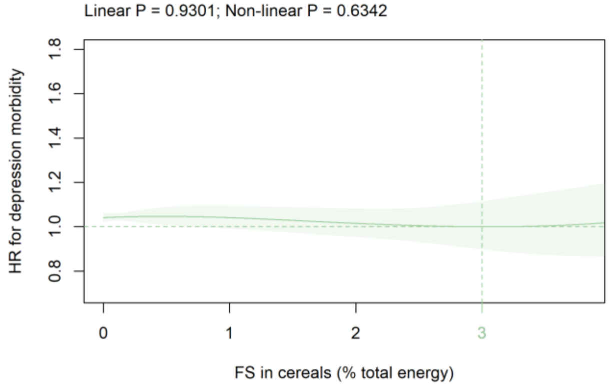

(k)

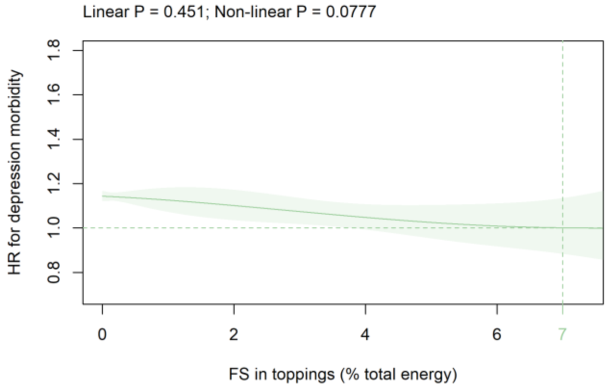

(l)

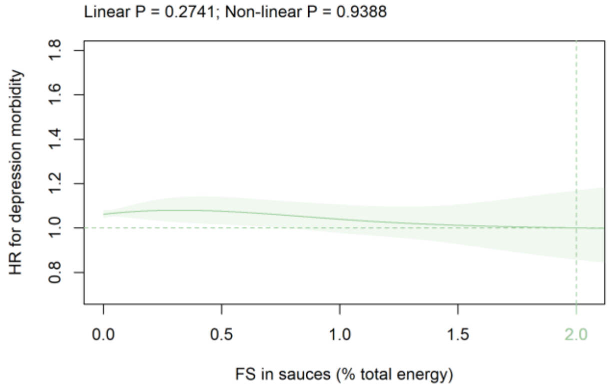

Online Resource 10 continued

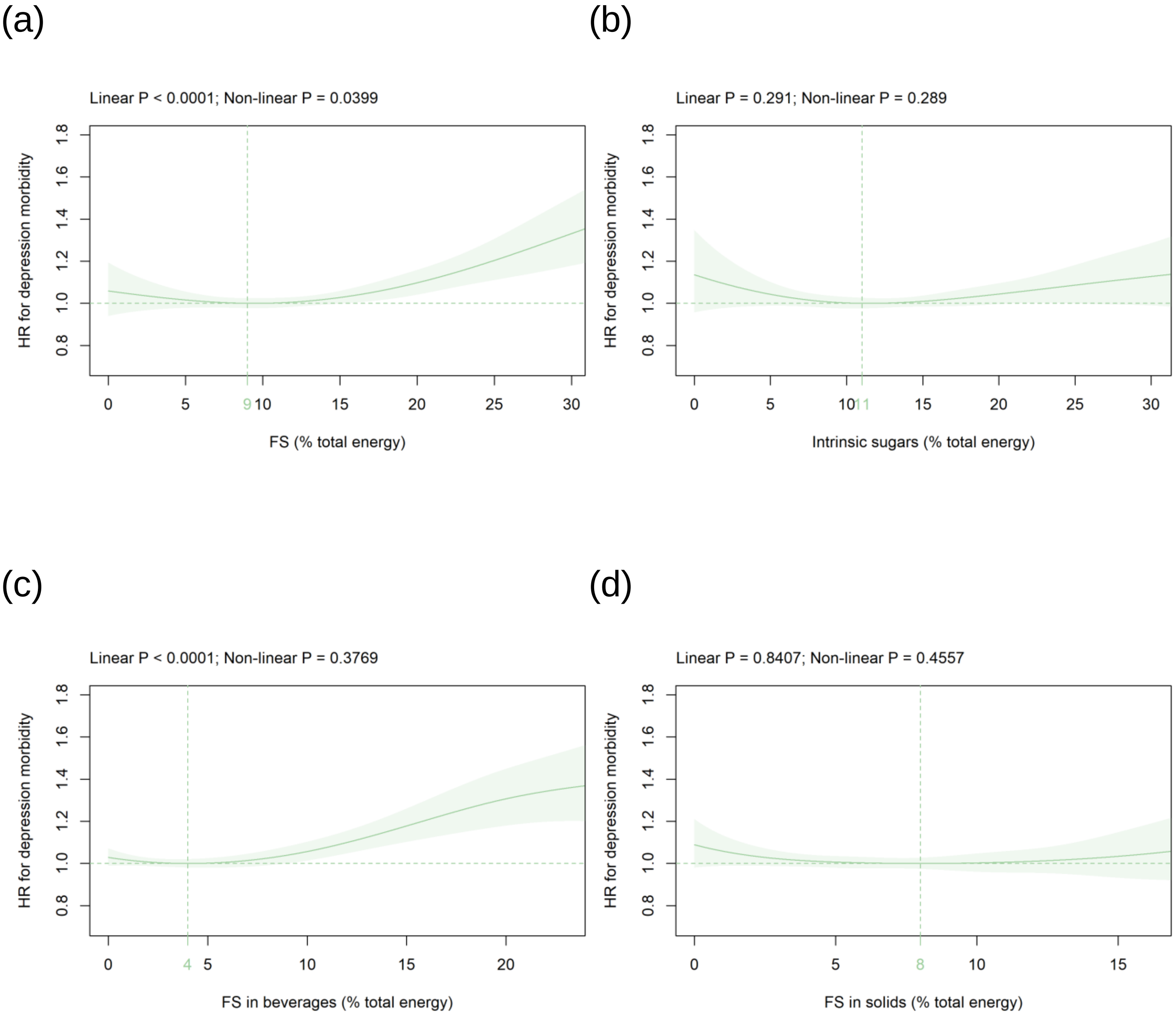

Online Resource 11

(e)

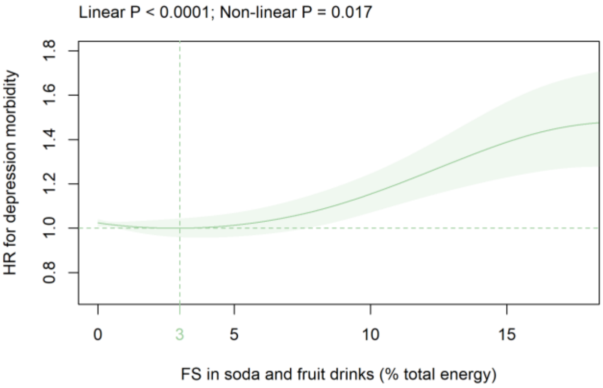

(f)

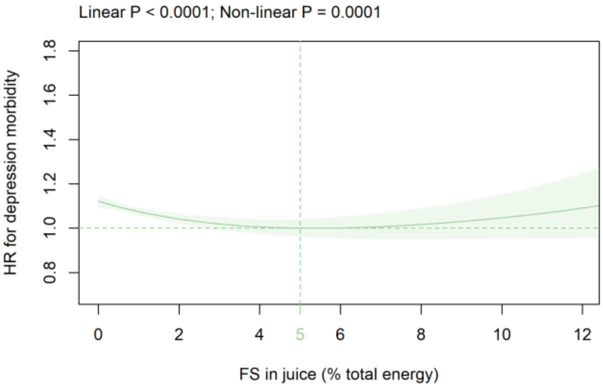

(g)

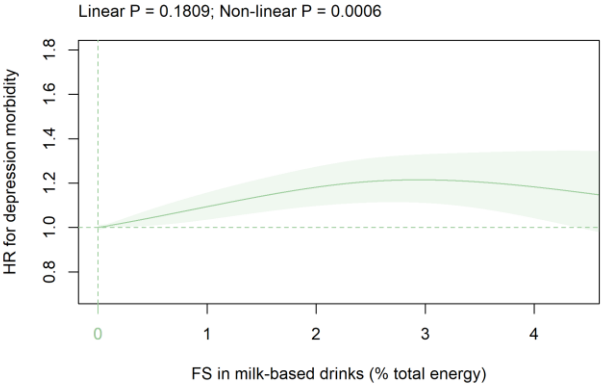

(h)

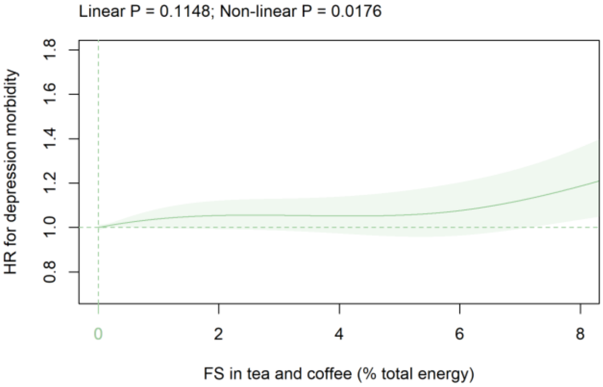

(i)

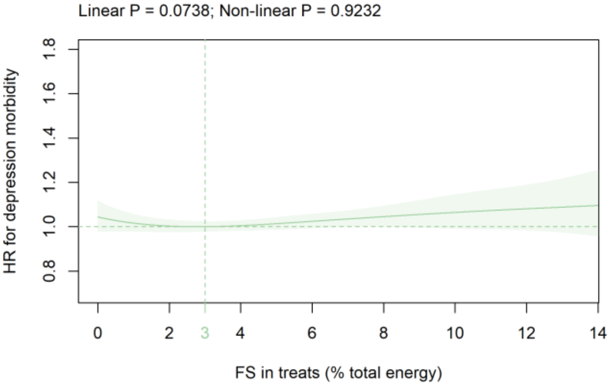

(j)

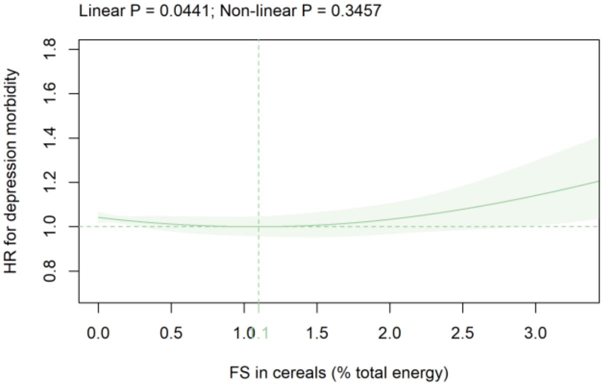

(k)

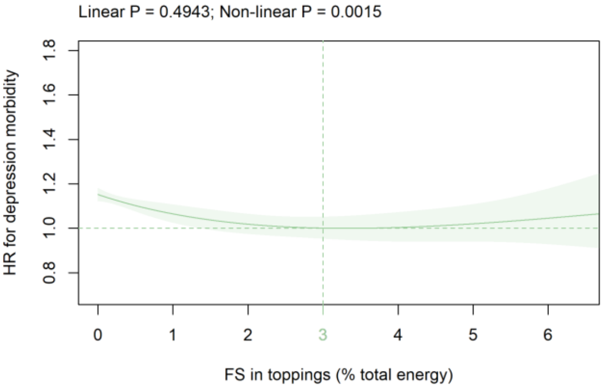

(l)

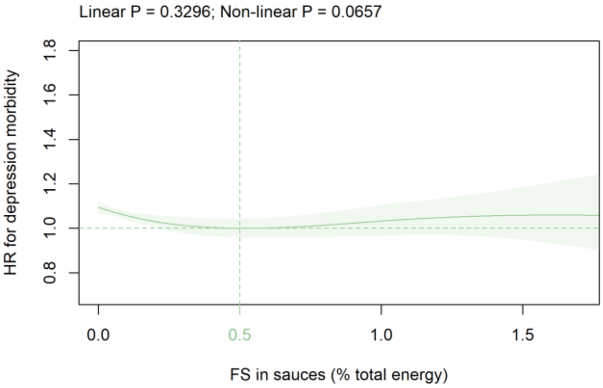

Online Resource 11 continued

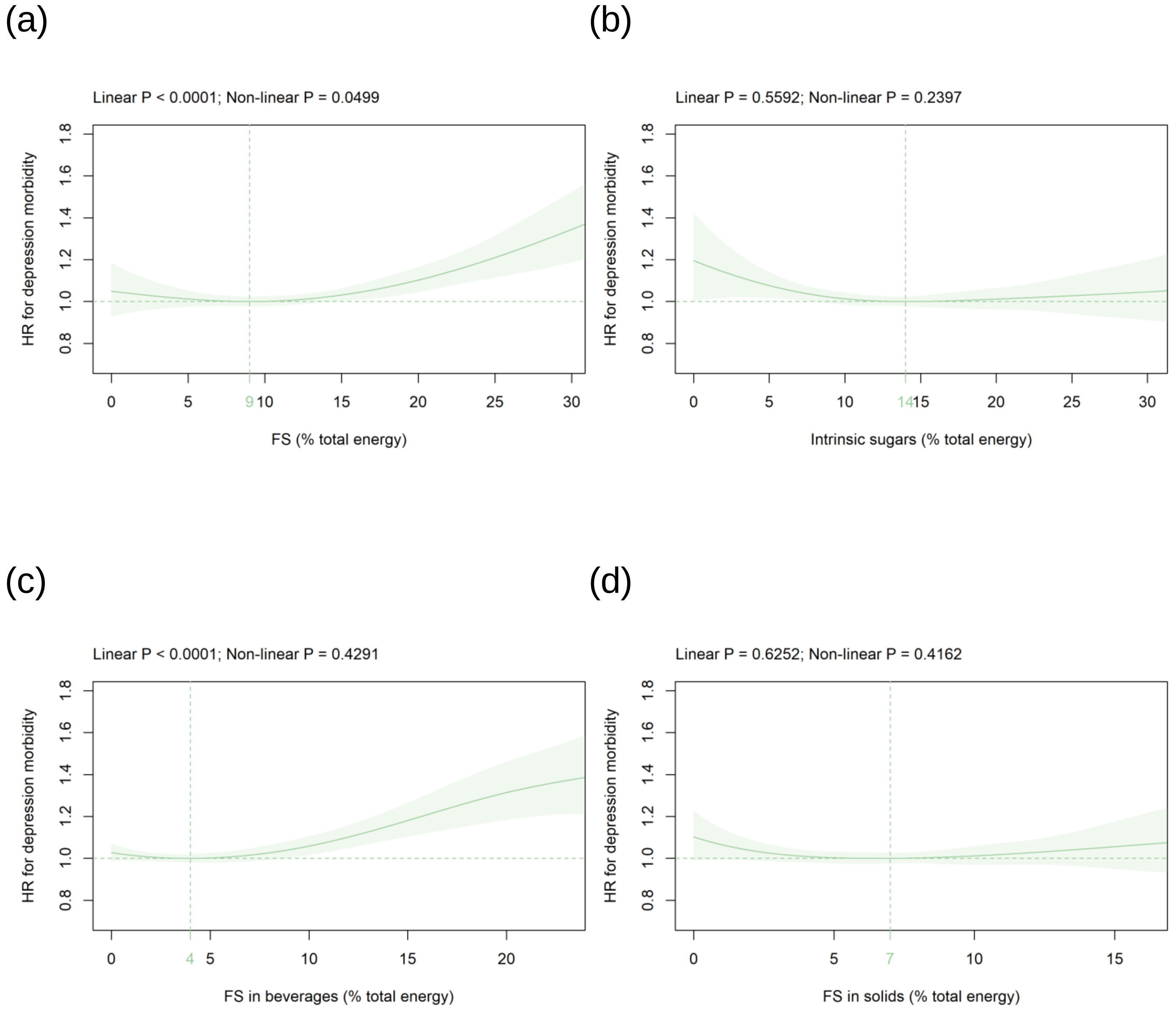

Online Resource 12

(e)

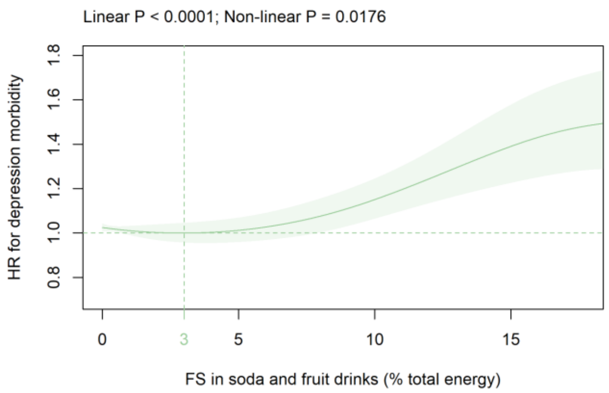

(f)

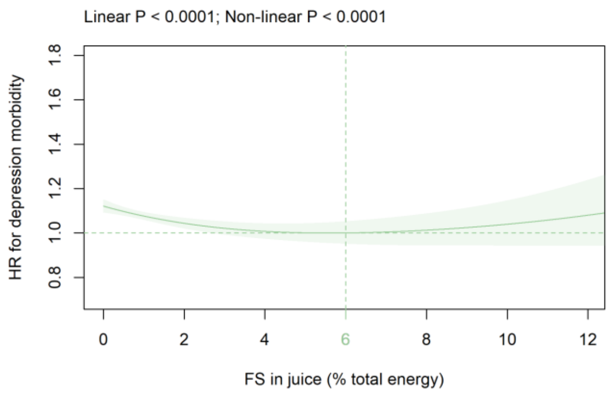

(g)

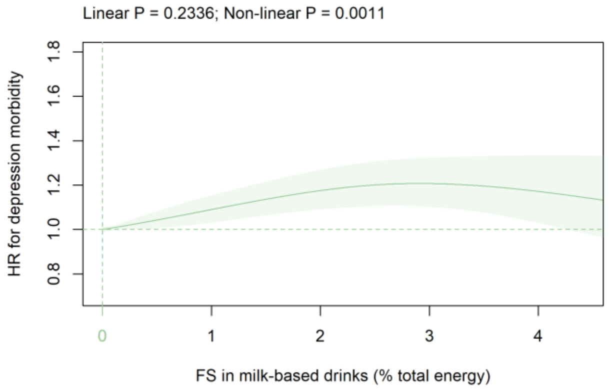

(h)

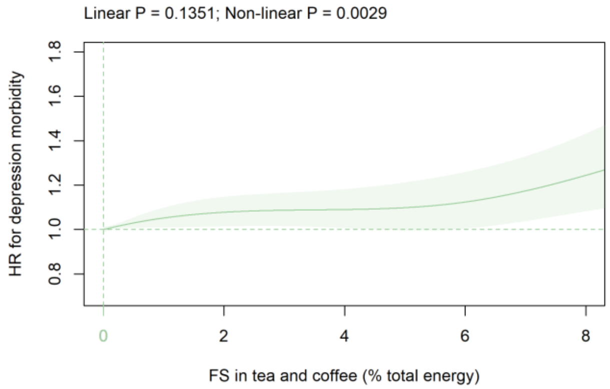

(i)

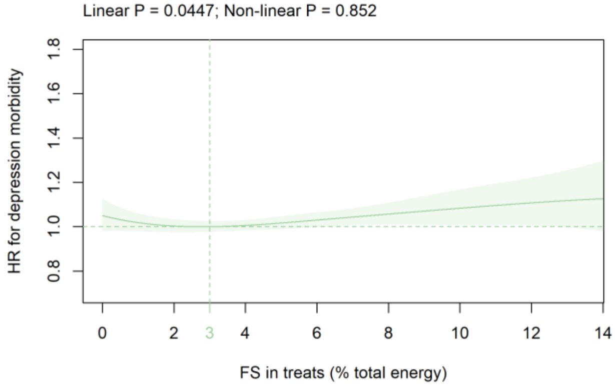

(j)

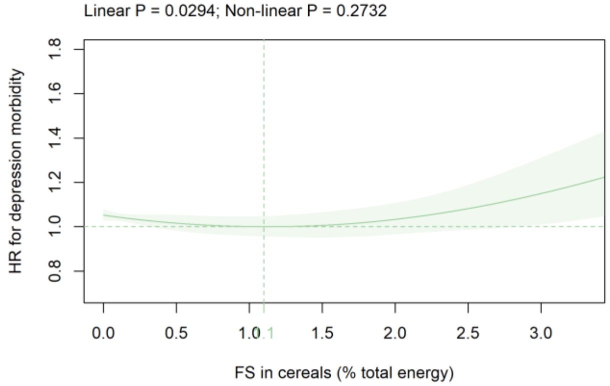

(k)

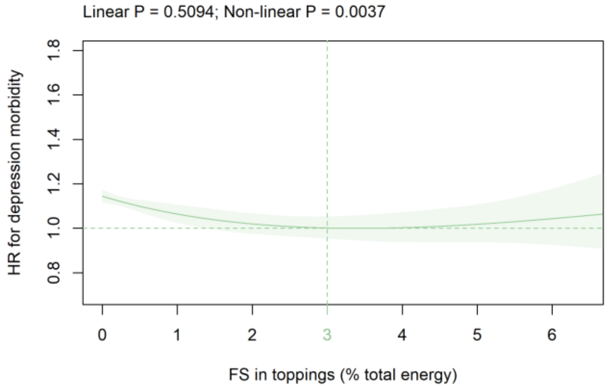

(l)

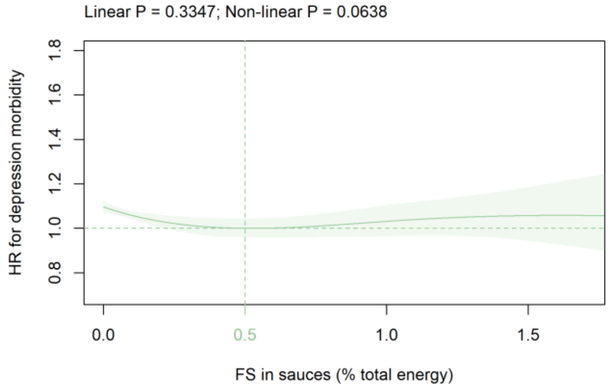

Online Resource 12 continued

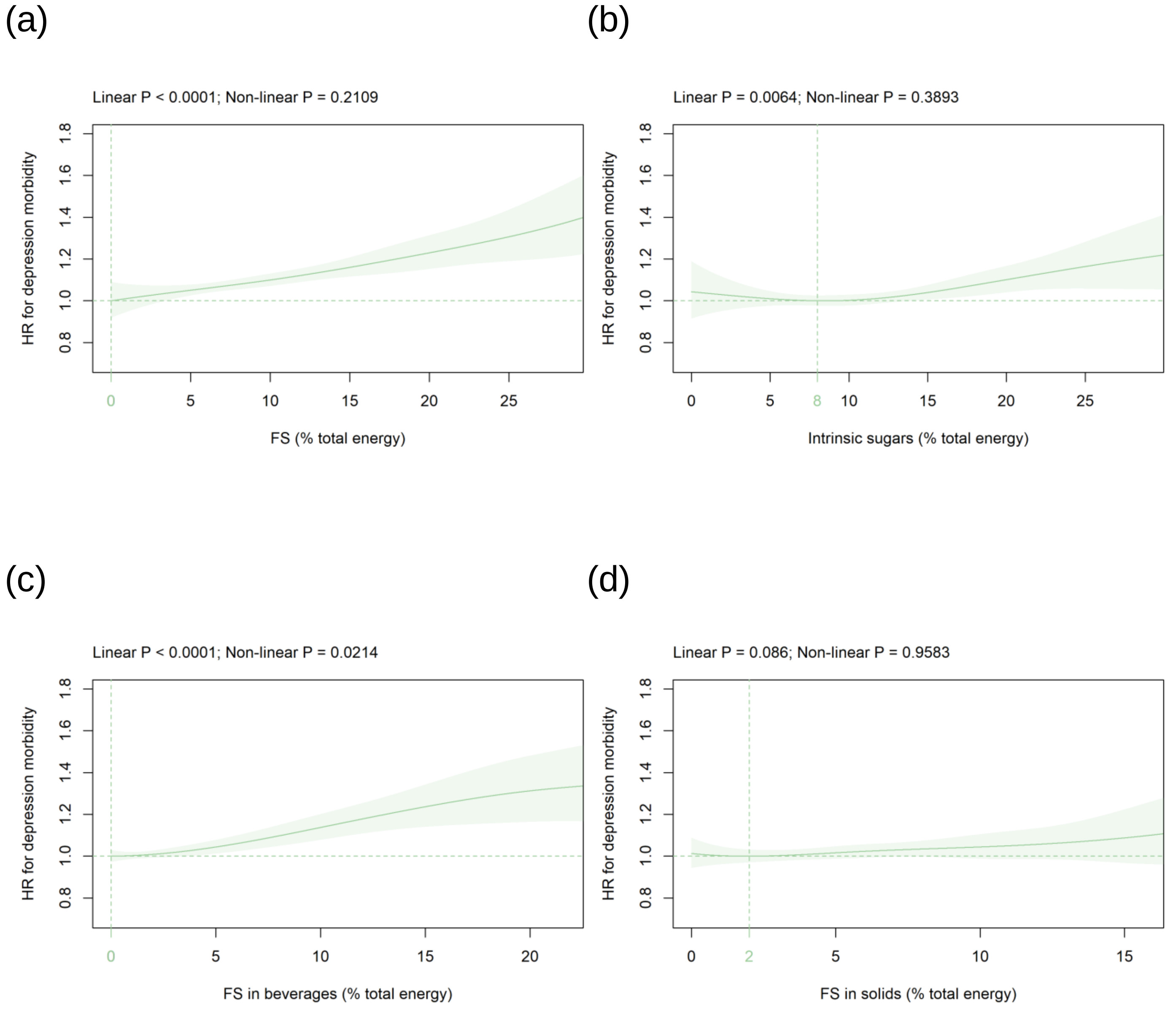

Online Resource 13

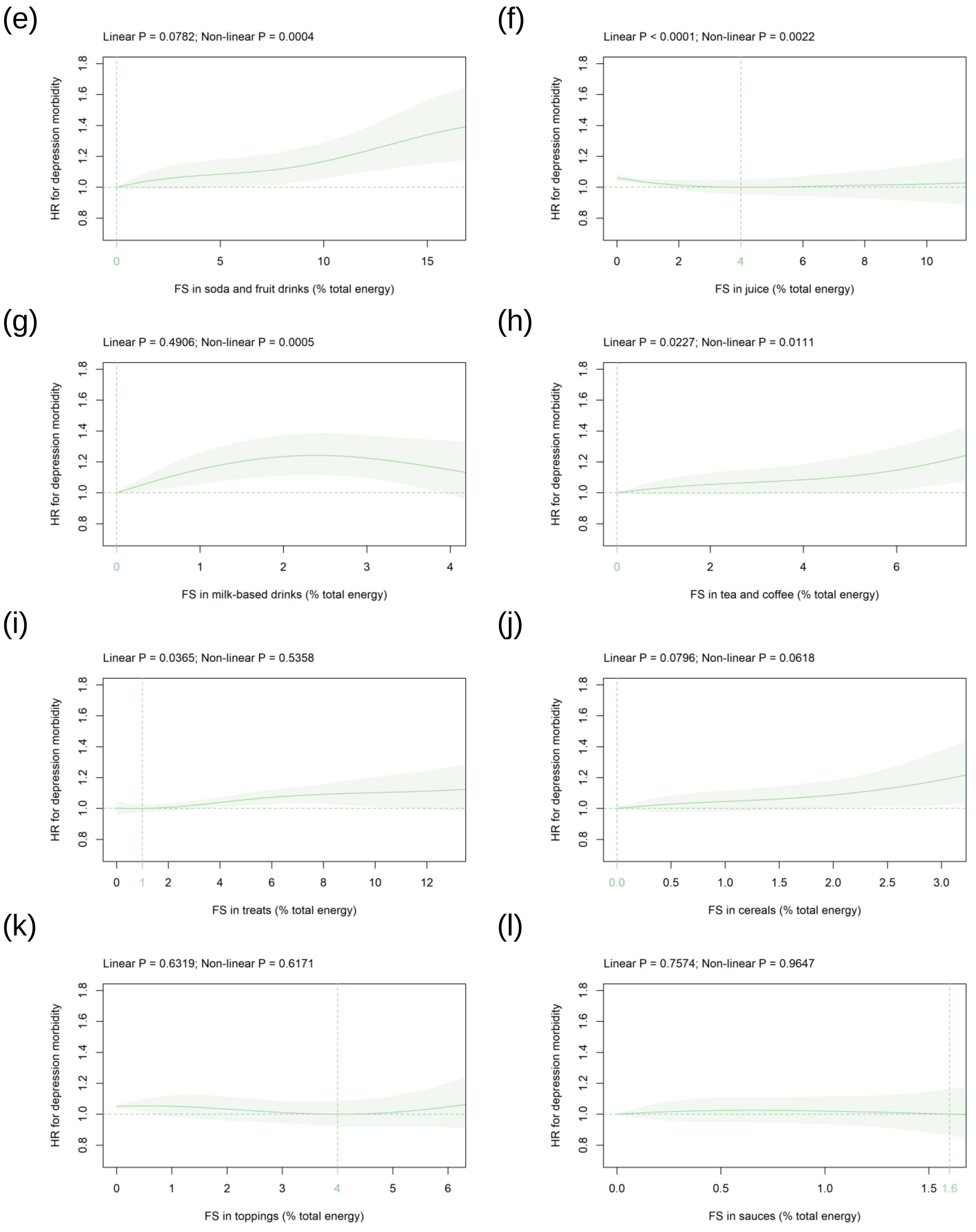

Online Resource 13 continued

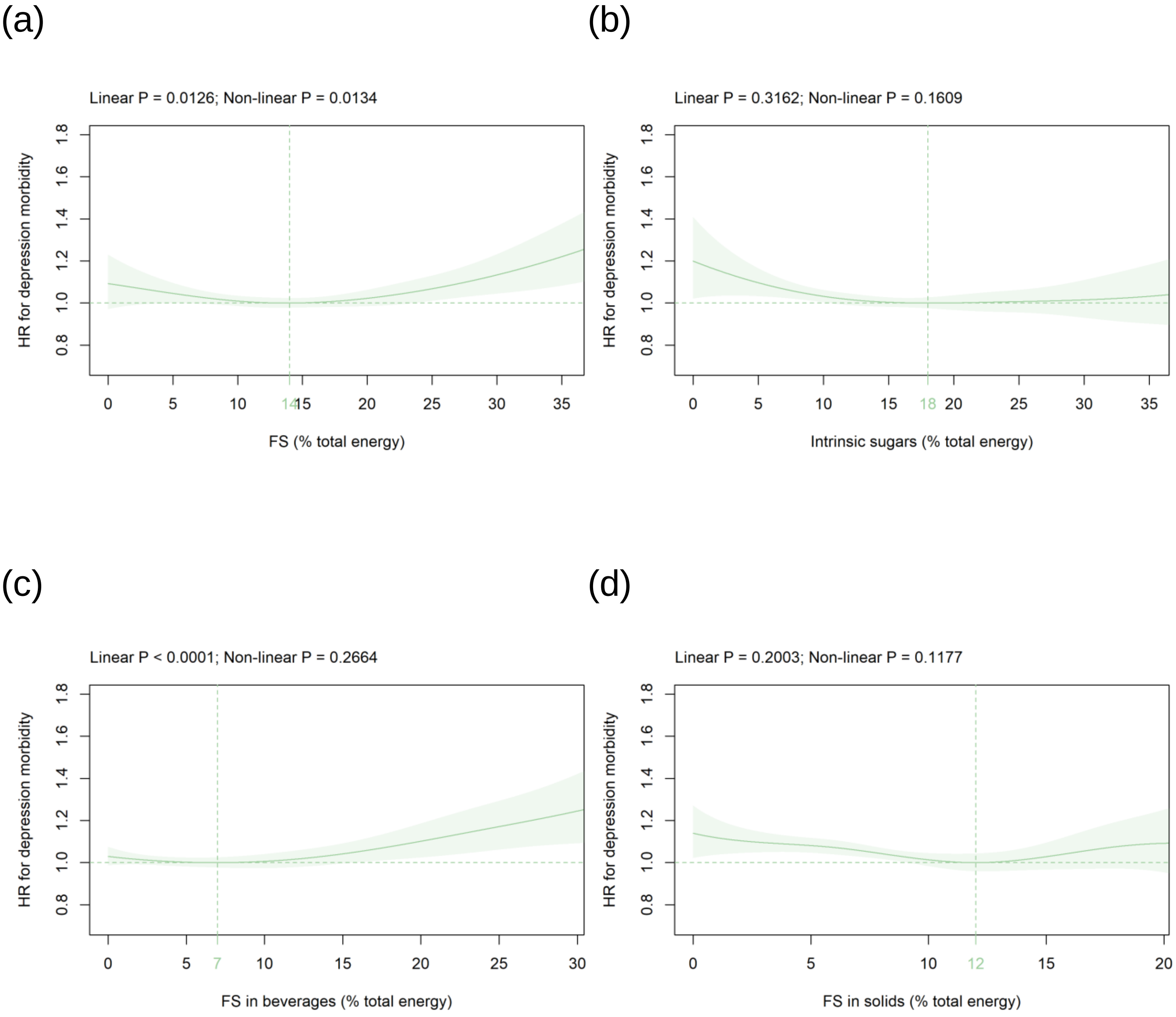

Online Resource 14

(e)

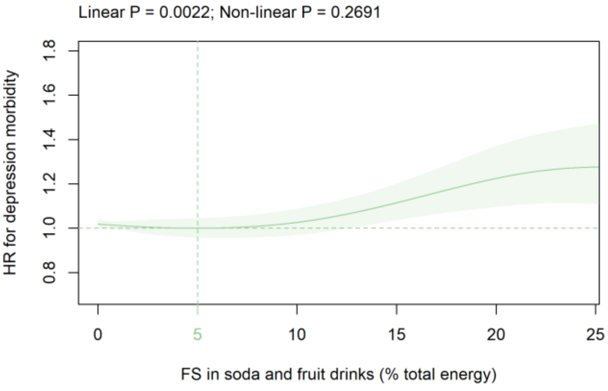

(f)

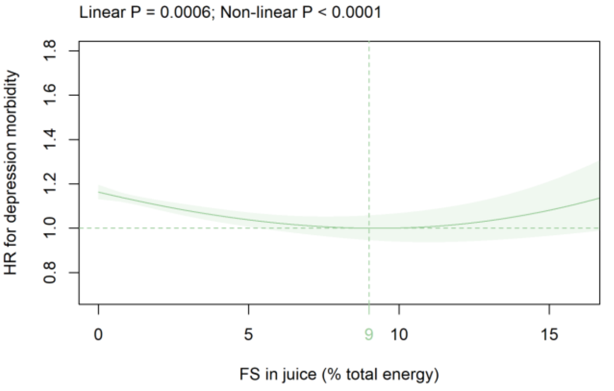

(g)

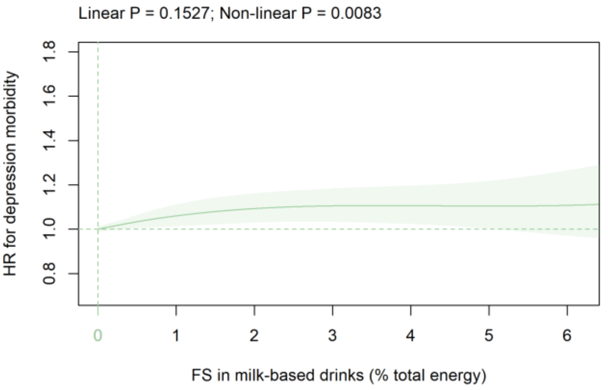

(h)

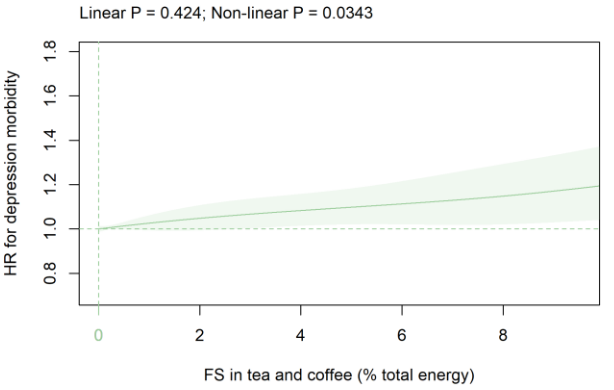

(i)

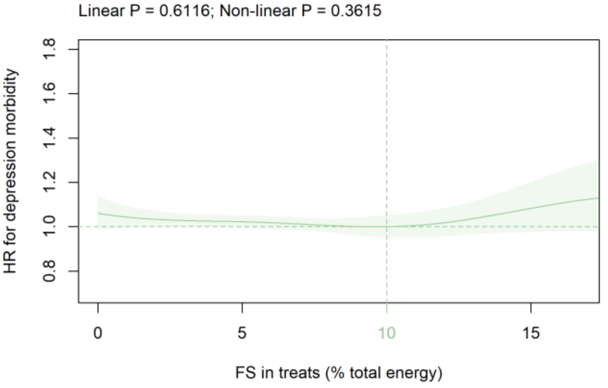

(j)

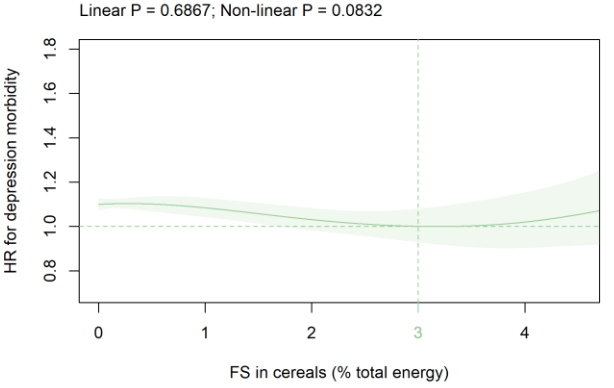

(k)

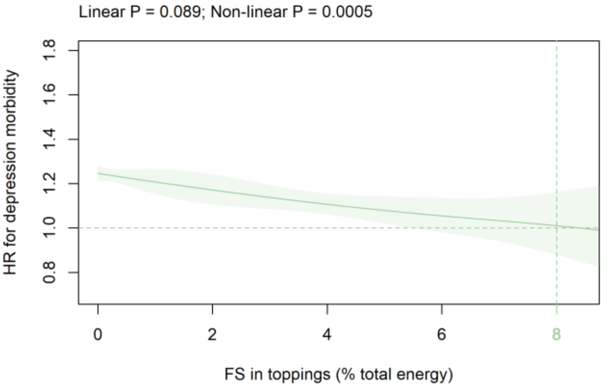

(l)

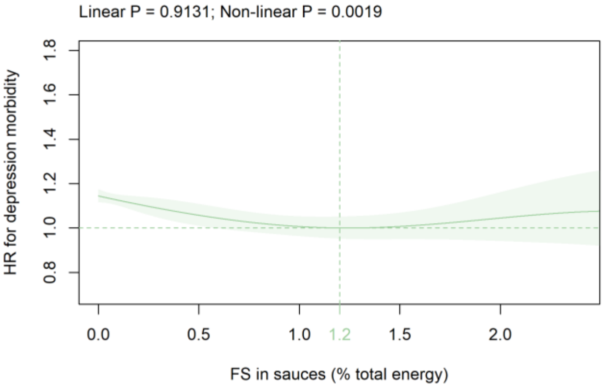

Online Resource 14 continued

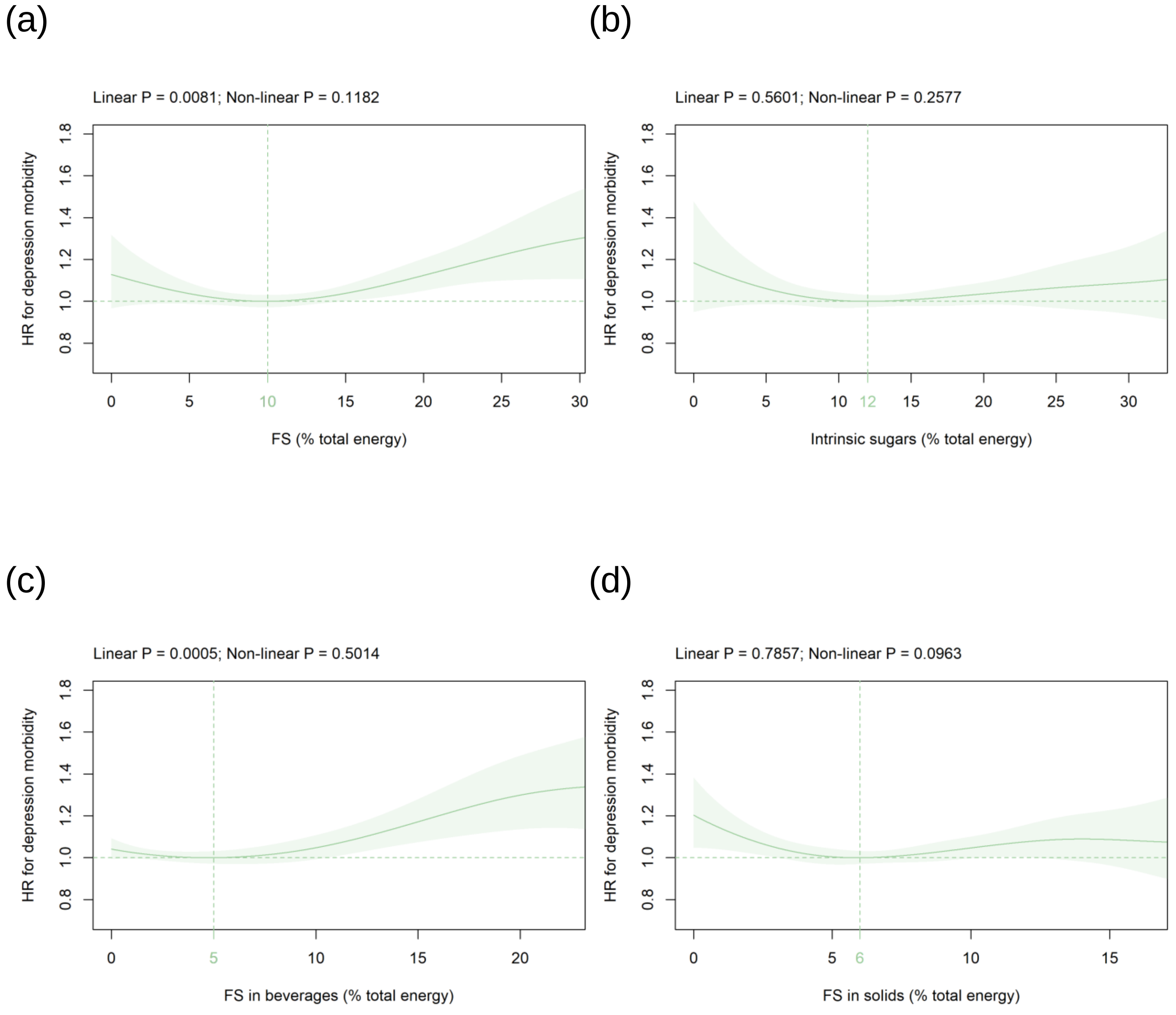

Online Resource 15

(e)

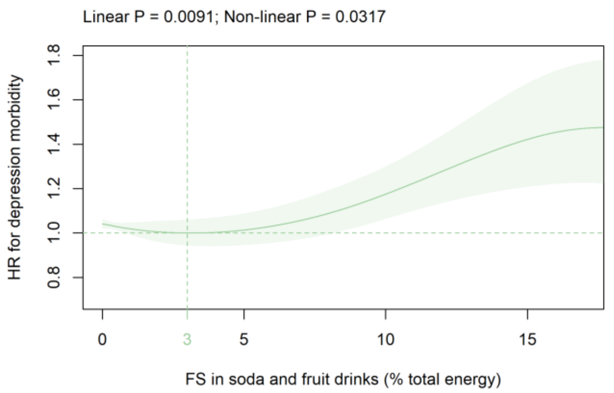

(f)

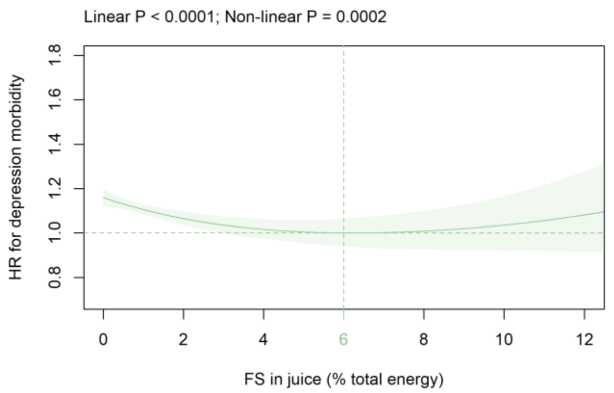

(g)

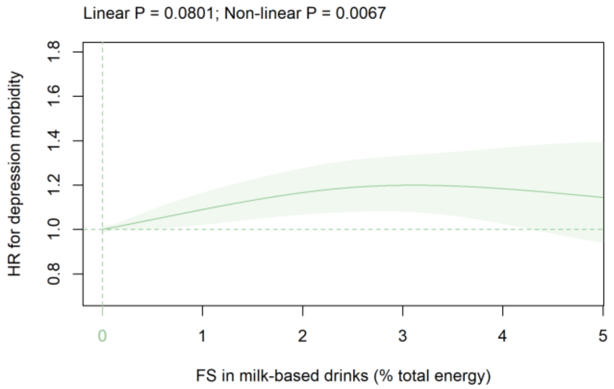

(h)

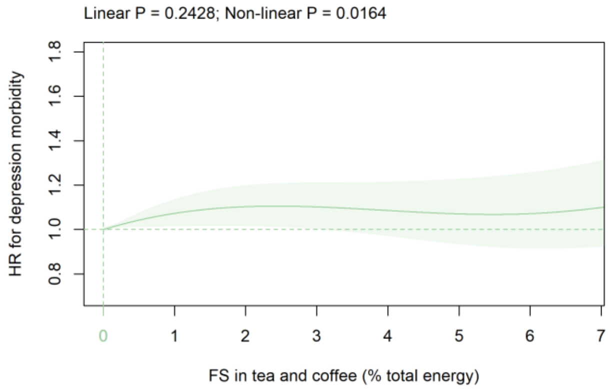

(i)

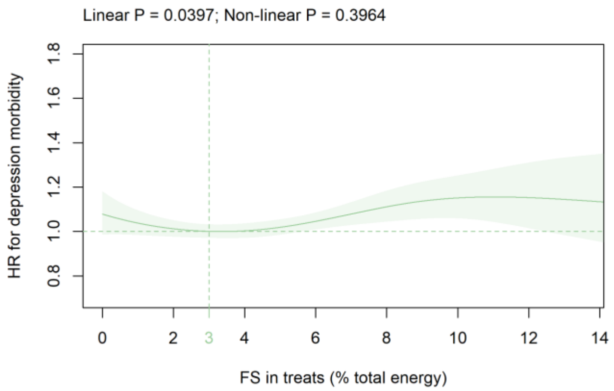

(j)

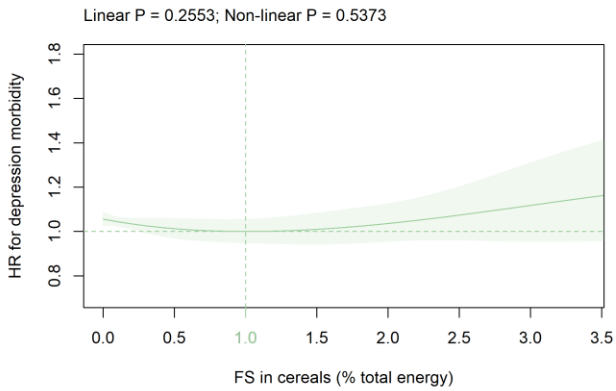

(k)

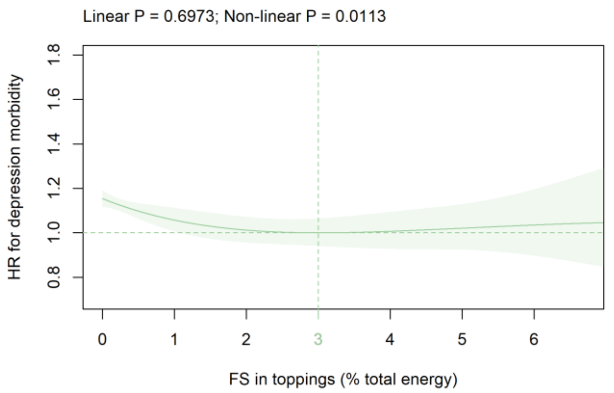

(l)

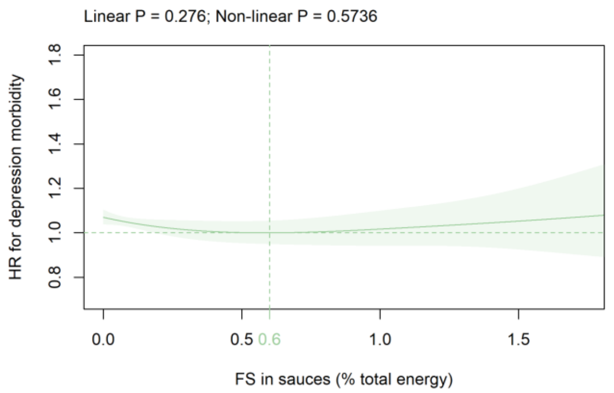

Online Resource 15 continued

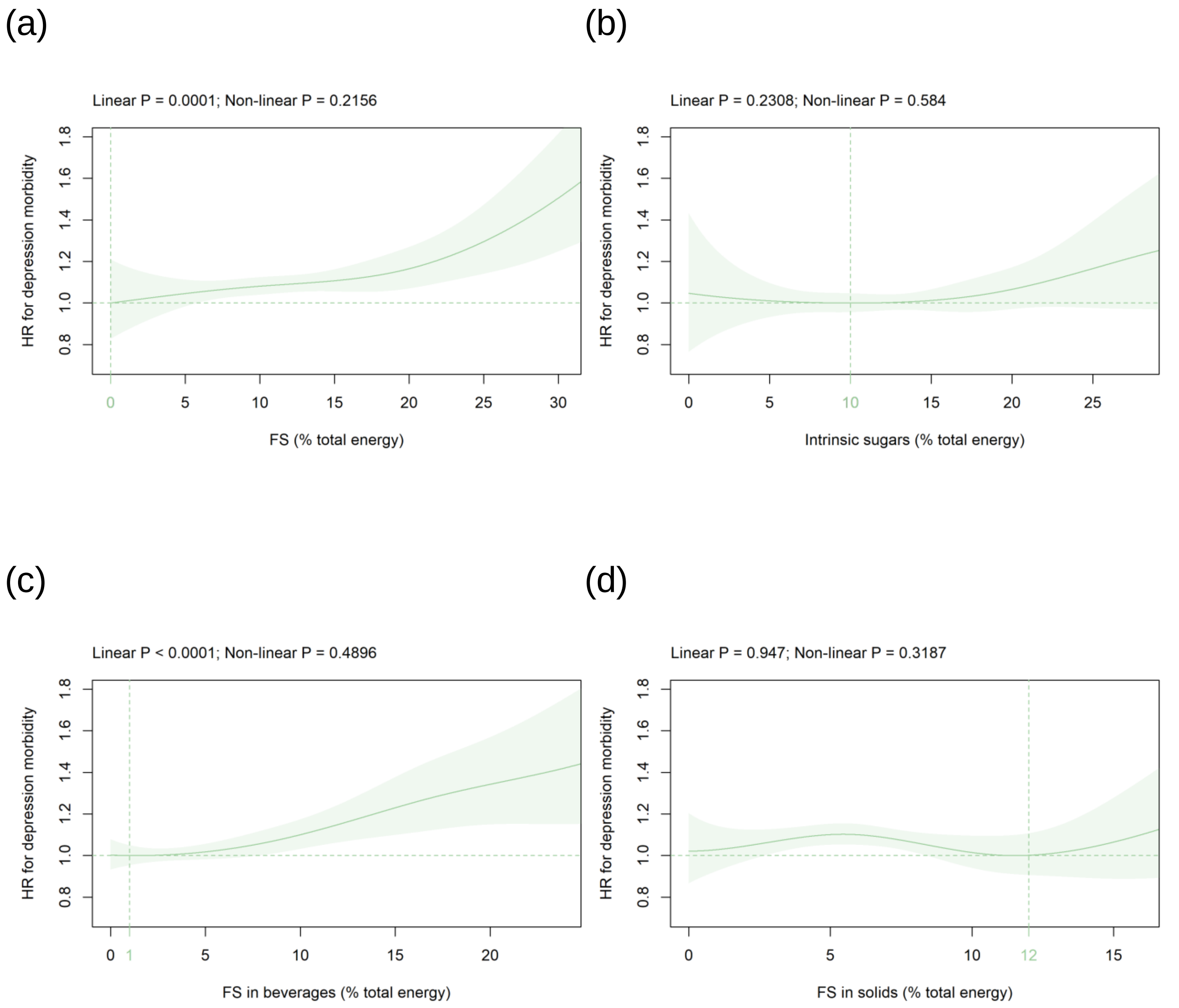

Online Resource 16

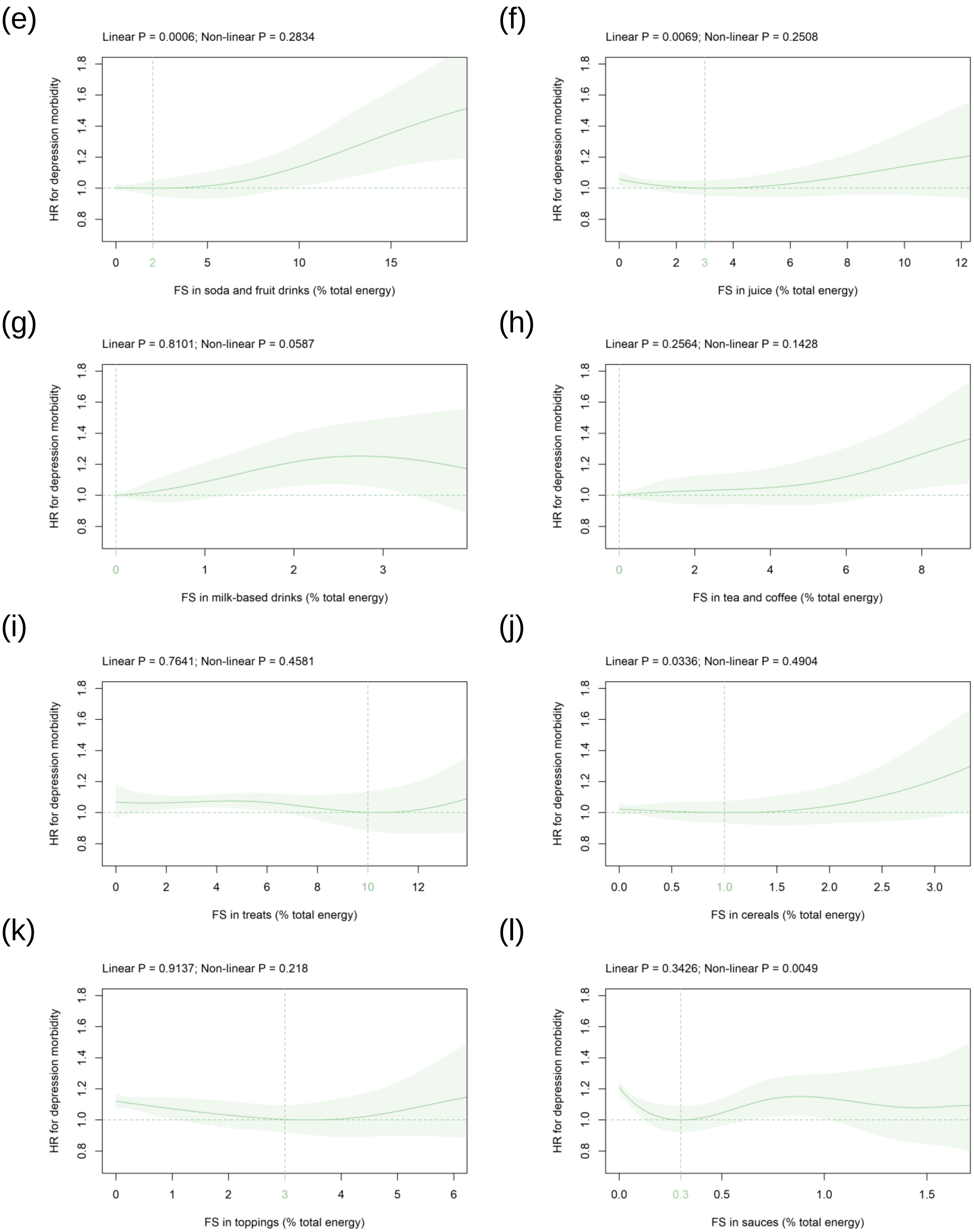

Online Resource 16 continued
